# Development of a plant growth-promoting bacterial EcoBiome derived from desert soil isolates

**DOI:** 10.1128/aem.00103-26

**Published:** 2026-04-13

**Authors:** Camila Albarrán-Cuitiño, Daniel E. Palma, Hugo González, José Antonio O'Brien, Mauricio González, Alexis Gaete

**Affiliations:** 1 Instituto Milenio Centro de Regulación del Genoma, Santiago, Chile; 2 Laboratorio de Bioinformática y Expresión Génica, Instituto de Nutrición y Tecnología de los Alimentos, Universidad de Chile 14655, Santiago, Chile; 3 Facultad de Ciencias Biológicas, Pontificia Universidad Católica de Chile 28033, Santiago, Chile; 4 Facultad de Agronomía y Sistemas Naturales, Pontificia Universidad Católica de Chile 28033, Santiago, Chile; The University of Tennessee Knoxville, Knoxville, Tennessee, USA

**Keywords:** plant growth-promoting, synthetic community, EcoBiome, soil bacterial community

## Abstract

The study of the application of plant growth-promoting (PGP) bacteria has increased due to their potential to enhance crop yield, improve nutrient acquisition, and increase plant tolerance to biotic and abiotic stresses. Developing synthetic microbial consortia represents a promising strategy, as it can enhance colonization success and functional synergy within the rhizosphere. In this study, we designed a stable EcoBiome derived from a synthetic community (SynCom) of 17 bacterial isolates obtained from three desert environments. We evaluated their PGP traits, including siderophore production, indoleacetic acid synthesis, phosphate solubilization, and nitrogen fixation. Using Oxford Nanopore Technologies (ONT), we sequenced 16S rRNA genes and tracked changes in relative abundance across successive subcultures under four temperature conditions. From this analysis, *Erwinia rhapontici* 1SR, *Pseudomonas yamanorum* RZ5, and *Plantibacter* sp. RU18 were identified as dominant isolates and subsequently selected to construct the EcoBiome. Functional characterization showed that these isolates exhibited complementary PGP traits, biofilm formation capacity, and tolerance to water deficit, both individually and jointly. These findings highlight the potential of desert-derived bacterial consortia as microbial resources for developing biostimulants to enhance plant resilience under environmental stress conditions.

**IMPORTANCE:**

This study demonstrates that desert-derived bacterial isolates can be rationally assembled into a stable and functionally complementary EcoBiome with plant growth-promoting traits. By integrating phenotypic screening with community dynamics across environmental conditions, we demonstrate that simplified bacterial consortia derived from synthetic communities (SynCom) can retain key ecological traits, such as persistence, biofilm formation, and water deficit tolerance. These findings expand our knowledge of how bacterial molecular resources adapted to desert environments can be harnessed as biostimulants and provide a framework for the development of EcoBiomes aimed at improving plant resilience to abiotic stress.

## INTRODUCTION

The plant-associated soil bacterial community has garnered increasing attention in recent years due to its potential to enhance plant resilience against a variety of biotic and abiotic stresses (1–3). As sessile organisms, plants depend primarily on their root systems for essential biological processes and interactions. Symbiotic association between plants and bacteria within the rhizosphere has been shown to improve adaptive capacity by modulating key physiological processes, including nutrient acquisition, pathogen control, growth promotion, and increased tolerance to environmental stressors (4–6). These effects, whether direct or indirect, significantly influence plant development. Such bacteria are classified as plant growth-promoting (PGP) organisms, with their role in plant health and productivity remaining an active field of research.

Given the beneficial properties of PGP bacteria, they have been proposed as alternatives to replace chemical fertilizers and be used as bioinoculants capable of alleviating adverse effects on plant crops (7, 8), particularly through exogenous inoculation with a synthetic community (SynCom). This strategy has been tested in diverse crop species, including tomato (*Solanum lycopersicum)* (9–12), wheat (*Triticum aestivum*) (13–15), and several fruit-bearing plants (16–21). These studies emphasize the critical role of microbial diversity in maintaining and enhancing ecosystem functions.

Recent research suggests that SynCom offers superior benefits over single-strain inoculants, primarily due to synergistic functional interactions that can more effectively promote plant growth. For example, inoculation with multiple PGP strains has been associated with improved plant biomass accumulation (22) and increased shoot and root growth in walnut seedlings (23). However, the design of effective and environmentally resilient SynCom remains challenging. Antagonistic interactions between microorganisms, competitive exclusion, and limited tolerance to environmental stress can compromise their stability and functionality (22, 24).

In this context, microbial communities native to desert soils exhibit a remarkable metabolic versatility and adaptive capacities compared to those from controlled environments or less stressful environments (25–28). These microorganisms can thrive under extreme conditions, including water scarcity, high salinity, and UV radiation (29, 30). Therefore, exploring desert-derived PGP bacteria represents a promising strategy to mitigate environmental stress in agriculture (31, 32). Furthermore, these bacteria often display a strong capacity to colonize root tissues and may even be vertically transmitted through seeds (33).

Several studies have focused on isolating soil bacteria from desert environments with PGP attributes (34–36), highlighting their potential as inoculants to enhance plant resilience under biotic and abiotic stresses (31, 37, 38). However, challenges remain in designing stable and functional SynCom from these native microorganisms. To address these limitations, rational design strategies are required to assemble microbial consortia that balance ecological compatibility with functional complementarity. The top-down approach begins at the community level and progresses toward specific bacterial members, allowing environmental and laboratory selection pressures to guide microbial assemblages toward defined biological processes. Conversely, the bottom-up approach starts with the identification of metabolic attributes, enabling prediction of how individual microorganisms may interact and contribute to targeted functions (39). Integrating these perspectives provides a structured basis for selecting compatible bacteria and developing SynCom with both ecological coherence and functional relevance.

Recently, a more specific approach derived from the concept of SynCom has been proposed, integrating not only PGP traits but also ecological principles, giving rise to the concept of EcoBiomes (40). This approach exploits the functional complementarity and ecological compatibility of microorganisms that co-occur within the same environment, with the aim of enhancing functional stability, bacterial persistence, and ecological coherence of the community. In this context, the design of an EcoBiome derived from desert environments represents a valuable source of microorganisms with adaptive traits that promote plant growth and confer tolerance to environmental stress.

Based on this conceptual framework, we adopt a combined top-down and bottom-up approach to develop a workflow for constructing a stable EcoBiome from a SynCom, selecting three bacterial isolates capable of persisting after successive subcultures and successfully competing with native microbial communities in desert soil.

Here, we isolated and characterized 17 bacterial strains from desert soils in Chile using molecular and culture-based approaches to evaluate their plant growth-promoting potential, including tolerance to water deficit. Together, these results define a functionally validated, desert-derived EcoBiome with PGP traits and water stress tolerance, contributing to the development of microbial resources for sustainable agriculture.

## MATERIALS AND METHODS

### Sampling sites and isolation of bacterial strains

**Fig 1:**
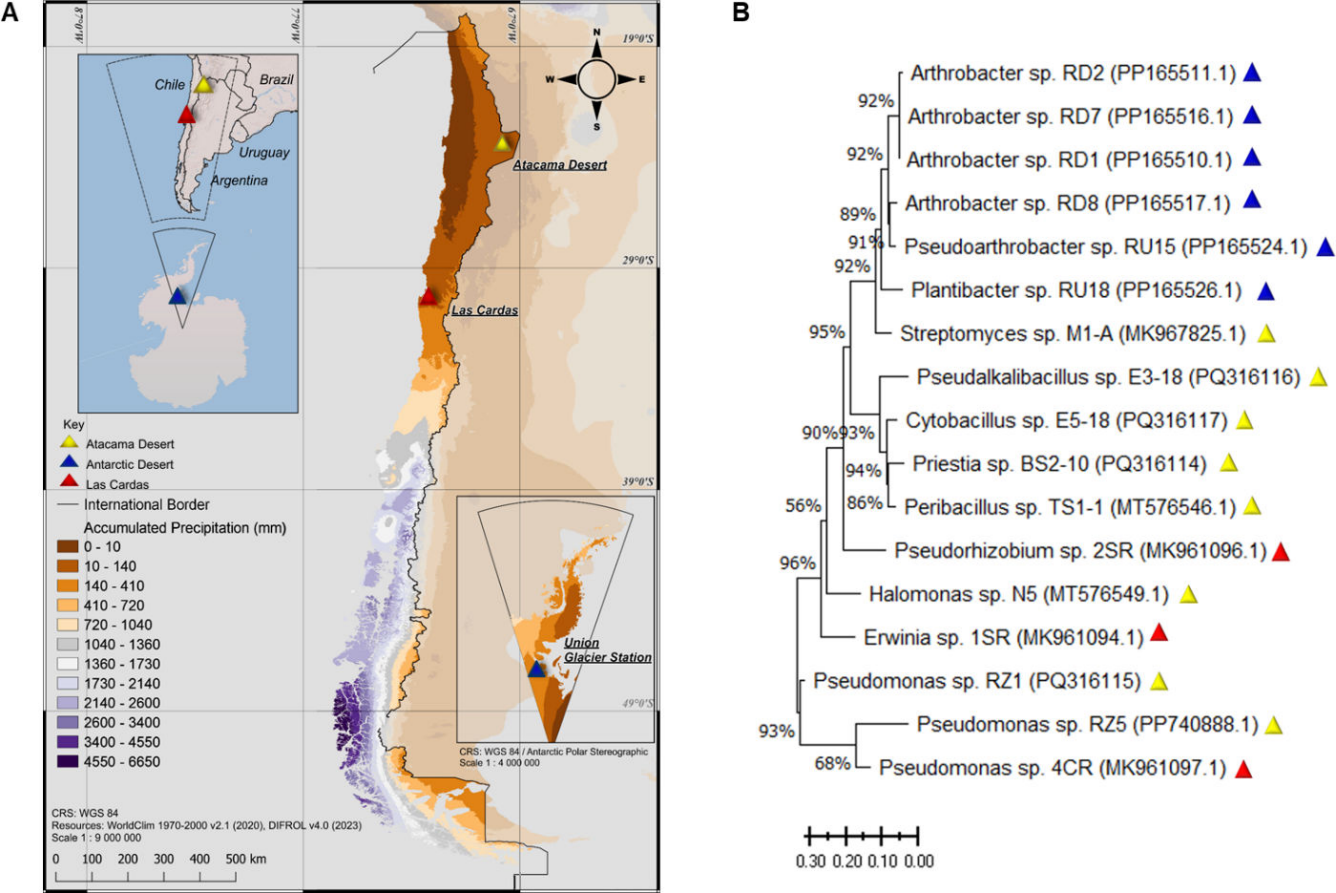
Geographic sites and phylogenetic classification of bacterial isolates used in this study. (**A**) Map of annual precipitation in Mainland Chile and Antarctica, defined as desert sites. (**B**) Phylogenetic tree based on 16S rRNA sequences of bacterial isolates from different desert environments. Colored triangles indicate the sampling sites: Antarctic Desert (blue), Las Cardas (red), and Atacama Desert (yellow). The GenBank code for each strain is identified in parentheses.

A total of 17 bacterial isolates were obtained from three sites classified as desert environments (Fig. 1A), based on the criterion of annual precipitation below 250 mm/year. Soil samples were collected from the Atacama Desert (23°21′06.9″S 67°50′01.0″W) in 2022, from the Experimental Station “Las Cardas” in the Limarí Valley, Coquimbo (30°15′06.6″S 71°15′23.8″W) in 2019, and from a site near the Union Glacier Station, Antarctic Desert (79°46′51.6″S 83°18′50.1″W) in 2022. At each location, surface soil was removed, and soil samples were collected from a depth of up to 20 cm. Samples were stored in sterile bags and kept refrigerated at 4°C until further processing.

### Bacterial isolation and taxonomic identification via 16S rRNA gene sequencing

Isolation was performed as per Mandakovic et al. (41). Briefly, 1 g of each soil sample was thoroughly mixed with 1 mL of 1× phosphate-buffered saline for 2 h using a Revolver Rotator (Labnet). After centrifugation at 8,000 rpm for 5 min, 100 µL of supernatant was plated onto a Luria Bertani (LB) agar and incubated at 10°C, 20°C, and 30°C for 72 h. Individual colonies were cultured in 24-well plates with 1 mL of LB medium, shaken at 160 rpm at 25°C for 24 h. The resulting cultures were stored at −80°C with glycerol in the internal microbial repository.

Genomic DNA was extracted using the DNeasy Blood & Tissue Kit (Qiagen) following the manufacturer’s specifications. The 16S rRNA gene was amplified with universal primers 27F/1492R at 10 µM each, 2 µL of genomic DNA, 12.5 µL of 2× GoTaq G2 Green Master Mix (Promega), and 8.5 µL of nuclease-free water, in a final volume of 25 µL. PCR conditions consisted of an initial denaturation at 95°C for 3 min, followed by 30 cycles of denaturation at 95°C for 30 s, annealing at 61°C for 30 s, and extension at 72°C for 90 s. A final extension step was performed at 72°C for 10 min.

Amplicon was sequenced using Sanger sequencing (Macrogen) and trimmed using the CLC Genomics Workbench (Qiagen). Taxonomic identification was conducted using the EzBioCloud 16S database, and all sequences were deposited in the National Center for Biotechnology Information (NCBI) GenBank database.

### *In vitro* characterization of plant growth-promoting traits

All the isolated bacteria were characterized using PGP biochemical assays, as described in Gaete et al. (35). Briefly, different culture media were used to assess four PGP traits and incubated at 30°C for 5 days.

Phosphate solubilization and nitrification were evaluated using media prepared according to the technical specifications provided by HiMedia. Pikovskaya agar (PKV) was used to assess phosphate solubilization (42), while a modified nitrification medium (NM) agar was used to test for nitrification (43). In both cases, the formation of a clear halo around bacterial colonies was interpreted as a positive result.

Siderophore production was assessed using chrome azurol S (CAS) agar medium, following the method described by Louden et al. (44). A color change from blue to orange, accompanied by the formation of a halo around the colony, was interpreted as a positive result. The diameter of the halo was recorded as a proportional measure of siderophore production.

The production of indole-3-acetic acid (IAA) was determined by a colorimetric assay using Salkowski reagent, following the method described by Mehmood et al. (45). A color change from yellow to red indicates IAA synthesis. The intensity of the color change was quantified by measuring absorbance and compared against a standard curve prepared with known IAA concentrations (0, 5, 10, 20, 50, and 100 µg/mL).

All statistical analyses were performed using GraphPad Prism software version 8.0.1 for Windows (GraphPad Software, La Jolla, CA, USA; www.graphpad.com).

### Design and evaluation of a bacterial synthetic co-culture

**Fig 2:**
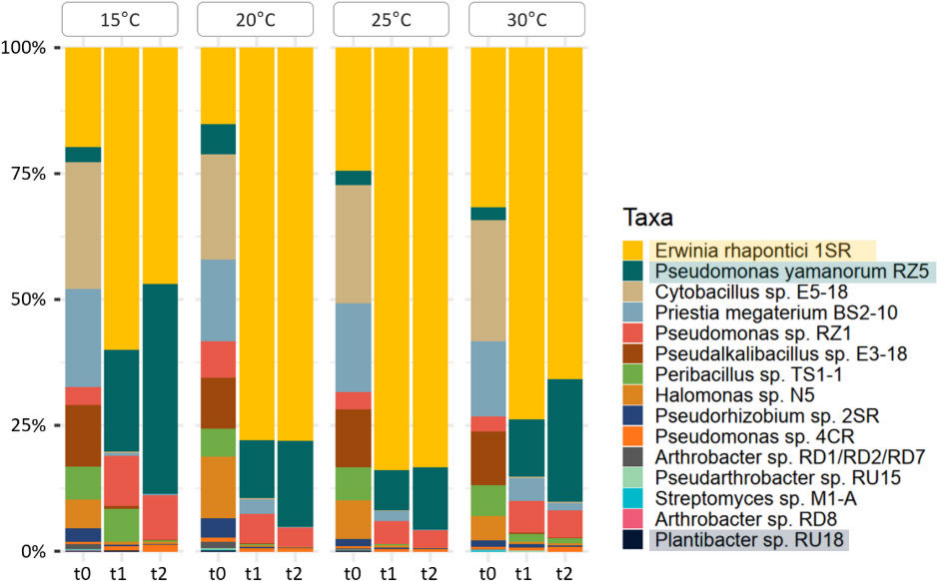
Relative abundance analysis of the synthetic community after two successive subcultures (t1 and t2) at four different agronomically relevant temperatures. Stacked bar plot of relative abundances based on 16S rRNA amplicon sequencing using Oxford Nanopore Technology.

Each bacterial isolate was independently cultured in LB medium at 15°C, 20°C, 25°C, and 30°C for 24 h at 160 rpm (Fig. S1). The optical density (OD_600_) of each culture was measured and adjusted to 0.1 using an Infinite 200 PRO NanoQuant spectrophotometer (Tecan) in a final volume of 10 mL. The culture was then divided into two equal fractions of 5 mL each. One fraction was used for genomic DNA extraction at time zero (t₀), and the second was used to evaluate the influence of temperature within an agronomic range (15°C–30°C) on bacterial growth in culture. Two subsequent subcultures (t₁ and t₂) were performed at 24-h intervals using a 1:10 dilution (500 µL of bacterial culture into 5 mL of fresh LB medium) (Fig. 2). After each incubation period, DNA was extracted from the synthetic community using the E.Z.N.A. Soil DNA Kit (Omega Bio-Tek), following the manufacturer’s instructions.

### Amplicon sequencing and relative abundance of the synthetic community

The extracted DNA was used to analyze the bacterial relative abundance through amplification of the 16S rRNA gene using the 16S Barcoding Kit SQK-RAB204 (Oxford Nanopore Technologies), following the manufacturer’s instructions. Sequencing was performed on a MinIon device version 1B (Oxford Nanopore Technologies) using an R9.4.1 flow cell.

Basecalling and demultiplexing of reads were conducted with Dorado version 0.5.2 (Oxford Nanopore Technologies), using the model dna_r9.4.1_e8_sup@v3.6. Filtering criteria included a minimum mean *Q*-score of 7, a minimum read length of 1,000 bp, and maximum read length of 2,000 bp. Read quality control was assessed using pycoQC version 2.5.2 (46). Consensus 16S rRNA sequences for the bacterial isolates were generated using NanoCLUST (47).

For each isolate, a single 16S rRNA sequence was selected according to the following priority order: (i) genomic-derived sequence, (ii) NanoCLUST consensus sequence, or (iii) Sanger-derived sequence. Selected sequences were aligned using Infernal version 1.1.4 (48) with the RF00177 covariance model from Rfam (49) and manually trimmed using AliView version 1.28 (50) to ensure comparable sequence lengths across isolates. Finally, Emu version 3.4.5 (51) was used to estimate the relative abundances of each isolate in the synthetic community using a custom database constructed from the selected 16S rRNA sequences.

The phylogenetic Neighbor-Joining tree was constructed using the 16S rRNA sequences of each bacterial isolate. The sequences were trimmed using the CLC software (QIAGEN Bioinformatics), multiple aligned and edited in the BioEdit biological sequence alignment editor, and finally clustered and visualized as a phylogenetic tree based on the neighbor-joining technique using 1,000 replicates as bootstrap to obtain statistical support, performed on MEGA software (52).

### Genomic DNA extraction, whole-genome sequencing, assembly, and annotation

Whole-genome sequencing was performed using the DNA extracted as described in “Amplicon sequencing and relative abundance of the synthetic community,” above. Library preparation was conducted using the Ligation Sequencing Kit (Oxford Nanopore Technologies), following the manufacturer’s protocol, and sequencing was performed on a MinIon device (version 1B) with an R9.4.1 flow cell. The BioProject is available at the NCBI under the number PRJNA999330. The genome identifiers (ID) of the bacteria are *Erwinia rhapontici* 1SR (GCA_050613375.1), *Plantibacter* sp. RU18 (GCA_050613355.1), and *Pseudomonas yamanorum* RZ5 (GCA_050613365.1).

Basecalling of Nanopore reads was performed with Dorado version 0.5.2 (Oxford Nanopore Technologies) using the model dna_r9.4.1_e8_sup@v3.6, applying the following criteria: a minimum mean *Q*-score of 7 and a minimum read length of 1,000 bp. Read quality was assessed using pycoQC version 2.5.2 (46). Consensus genome assemblies were generated with Trycycler version 0.5.4, based on 12 individual assemblies from read subsets: 4 generated using Raven version 1.8.1 (53), 4 with Flye version 2.9.2 (54), and 4 using the combination of Minimap2 version 2.26 (55), Miniasm version 0.3 (56), and Minipolish (57). The resulting consensus contigs were polished using Medaka (Oxford Nanopore Technologies). Circular contigs were rotated to start at the dnaA gene, when present, using the fixstart function of Circlator version 1.5.5 (58). Genome completeness and contamination were assessed using CheckM version 1.2.2 (59) with the lineage workflow. Taxonomic classification was performed using GTDB-Tk version 2.3.2 (60), employing GTDB release 214 (61).

Genome annotation was conducted with Bakta version 1.8.1 (62), using database version 5.0. Circular genome plots were generated using Circos (63). Genome sequences were uploaded to the Type (Strain) Genome Server (TYGS) for a whole-genome-based taxonomic analysis (64), incorporating recent methodological improvements (65). Nomenclature information, synonymy, and relevant taxonomic literature were retrieved via TYGS’s sister database, the List of Prokaryotic names with Standing in Nomenclature (65). A phylogenetic tree was constructed using FastME 2.1.6.1 (66), based on genome BLAST distance phylogeny (GBDP) distances calculated from genome sequences. Branch lengths were scaled according to the GBDP distance formula d5. Bootstrap support values (>60%) from 100 replications are indicated above the branches, with an average support of 94.9%. The tree was rooted at the midpoint (67).

### Genome-based prediction of metabolic pathways and secondary metabolites

A comprehensive analysis of metabolic pathways and secondary metabolite biosynthesis was conducted using KofamKOALA and antiSMASH, respectively. KofamKOALA tool utilizes a reliability-based K-value scoring system (68) to compare protein sequences with Kyoto Encyclopedia of Genes and Genomes (KEGG) orthologs, allowing the identification of metabolic pathways present in the bacterial genomes. Particular emphasis was placed on detecting KEGG orthologs associated with siderophore production, tolerance to heat and water deficit, IAA biosynthesis, nitrogen fixation, 1-aminocyclopropane-1-carboxylate deaminase activity, and iron and phosphate uptake, as described by Gaete et al. (35) and Chávez-Ávila et al. (69). The analysis was also extended to identify genes associated with biofilm formation, as well as the biosynthesis of pyocyanin and pyoverdine.

The biosynthetic potential for specialized metabolites was assessed using antiSMASH version 7.0 (70) for bacterial genomes. This tool identifies biosynthetic gene clusters, and analyses were conducted using the “relaxed” stringency parameter to maximize the detection of potential clusters.

### EcoBiome assembly and compatibility assessment

The EcoBiome was constructed following the definition and selection criteria described by Mousa et al. (40). Briefly, the selection process considered preserving the representativeness of the native bacterial communities of each desert environment and presenting the highest number of plant growth-promoting traits. Additionally, we applied another criterion based on high relative abundance in co-culture conditions after successive subcultures. Thus, three bacterial isolates were selected: *Erwinia rhapontici* 1SR, *Pseudomonas yamanorum* RZ5, and *Plantibacter* sp. RU18, which were used to evaluate the establishment of the EcoBiome.

Compatibility between the selected isolates was assessed through competition and attraction assays. The competition activity was performed on LB agar medium following the procedure described by Pérez-y-Terrón et al. (71), while the attraction activity was carried out according to Berendsen et al. (72). For the competition assay, 100 µL of each isolate was inoculated onto the surface of a Petri dish and allowed to dry for 5 min. Subsequently, 10 µL of a second isolate was inoculated at the same location. Inhibition was indicated by the formation of a clear halo around the bacterial culture. The attraction assay involved inoculating 1 µL of each individual isolate diagonally in V-shape rows. The assay was considered positive when the bacterial colonies exhibited chemotactic behavior, evidenced by changes in their circular morphology as they moved closer together. Both assays were conducted at 15°C, 20°C, 25°C, and 30°C, with evaluation performed after 24 and 48 h of incubation.

### Characterization of *in vitro* plant growth-promoting traits and stress tolerance in the EcoBiome

*In vitro* evaluation of PGP traits for the EcoBiome was performed as described in “*In vitro* characterization of plant growth-promoting traits,” above, with modifications. Briefly, the assay was conducted using individual isolates, pairwise combinations, and the complete EcoBiome by inoculating 10 µL of each isolate as required.

The biofilm production capacity and water deficit tolerance of the EcoBiome were evaluated following the protocols described by Chávez-Avila et al. (69). For biofilm formation, isolates were cultured in Eppendorf tubes for 6 days in static conditions. Subsequently, 0.3% crystal violet was added and incubated at room temperature for 15 min. The supernatant was discarded, and the tubes were washed with distilled water. The dye adhering to the tube walls was then solubilized with 1 mL of 100% ethanol, and absorbance was measured at 590 nm.

The water deficit tolerance assay was performed on LB agar plates supplemented with 5% and 10% polyethylene glycol 8000, and the plates were incubated for 24 h. In all assays, bacterial suspensions were adjusted to an optical density at 600 nm (OD_600_) of 0.1 and incubated at four temperatures: 15°C, 20°C, 25°C, and 30°C.

### Characterization of colonization and persistence of the EcoBiome in the soil

The EcoBiome soil inoculation assay was performed using a bacterial consortium composed of *Erwinia rhapontici* 1SR, *Pseudomonas yamanorum* RZ5, and *Plantibacter* sp. RU18. Each strain was independently cultured for 24 h in LB medium at 20°C under constant agitation at 180 rpm. Subsequently, the cultures were adjusted to 10² cells/mL and combined to obtain a final volume of 8 mL of the EcoBiome inoculum.

Non-sterile soil collected from the Atacama Desert in April 2023 was used as the substrate. Experiments were conducted in triplicate in Petri dishes, using 50 g of soil per replicate. Each soil sample was inoculated with 500 µL of the EcoBiome, while the negative control consisted of non-inoculated soil.

Additionally, the effect of EcoBiome inoculation was evaluated under different soil moisture conditions, considering four treatments: desert soil (without water addition) and soils adjusted to 5%, 10%, and 15% moisture by adding 2.5, 5, and 7.5 mL of sterile water, respectively.

The assay was maintained for 7 days at room temperature, after which total DNA was extracted from 500 mg of soil using the E.Z.N.A. Soil DNA Kit (Omega Bio-tek), following the manufacturer’s instructions.

DNAs were used to determine the taxonomic diversity and relative abundances of bacteria through whole-genome sequencing of the 16S rRNA gene using Oxford Nanopore Technology, as described in “Amplicon sequencing and relative abundance of the synthetic community,” above.

## RESULTS

### Taxonomic identification and *in vitro* characterization of plant growth-promoting traits

A total of 17 bacterial isolates were obtained from three Chilean desert soils (Fig. 2): 8 from the Atacama Desert, 6 from the Antarctic continent, and 3 from Las Cardas, Coquimbo Region. In terms of taxonomic diversity, the Atacama Desert isolates were classified into seven different genera, while the Antarctic isolates belonged to only three genera. The three isolates from Las Cardas each represented different genera.

The phylogenetic tree constructed using the 16S rRNA gene sequences (Fig. 1B) showed that the Antarctic Desert isolates formed a monophyletic clade with 91% bootstrap support. All members of this clade belonged to the order Micrococcales, with a well-supported internal group of closely related sequences from the genera *Arthrobacter* and *Pseudoarthrobacter*. A phylogenetically close isolate, *Streptomyces* sp. M1-A, was obtained from the Atacama Desert.

Another observed clade is supported by a 93% bootstrap and includes the bacterial genera *Pseudalkalibacillus*, *Cytobacillus*, *Priestia,* and *Peribacillus*, all included in the order *Bacillales* and isolated from the soil of the Atacama Desert.

Finally, another clade, with 90% bootstrap support, corresponded to the phylum Pseudomonadota and included three Atacama Desert and three Las Cardas isolates.

Subsequently, the PGP traits of each isolate were analyzed using specific culture media (Table 1), including siderophore production (CAS), phosphate solubilization (PKV), nitrification (NM), and indole-3-acetic acid, expressed in µg/mL. The results showed that 11 bacterial isolates exhibited a positive response for at least one PGP trait, specifically IAA production, with concentrations ranging from 0.99 to 84.024 µg/mL. Notably, the *Plantibacter* sp. RU18 isolate (from the Antarctic Desert) demonstrated the highest IAA production. Several isolates exhibited multiple PGP traits. *Streptomyces* sp. M1-A and *Pseudorhizobium* sp. 2SR tested positive for both siderophore production (CAS) and IAA. *Pseudomonas* sp. 4CR showed positive activity in PKV, NM, and IAA production. Meanwhile, *Pseudomonas* sp. RZ1, *Pseudomonas* sp. RZ5, and *Erwinia* sp. 1SR were positive for all four PGP traits: CAS, PKV, NM, and IAA.

**TABLE 1:** Summary of PGP trait characterization assays^*a*^

Location	Isolate	Genus	CAS			PKV			NM			IAA (µg/mL)			
			I	II	III	I	II	III	I	II	III	I	II	III	Avg.
Atacama Desert	BS2-10	*Priestia* sp.	−^*b*^	−	−	−	−	−	−	−	−	7.276	4.834	6.522	6.211
	RZ1	*Pseudomonas* sp.	++++	++++	++++	+	++	++	++	++	++	2.429	3.533	4.860	3.607
	RZ5	*Pseudomonas* sp.	++++	++++	++++	+++	++++	++++	++++	++++	++++	4.922	1.872	1.707	2.834
	M1-A	*Streptomyces* sp.	+	+	+	−	−	−	−	−	−	1.359	0.902	0.440	0.900
	TS1-1	*Peribacillus* sp.	−	−	−	−	−	−	−	−	−	6.468	7.058	6.082	6.536
	N5	*Halomonas* sp.	−	−	−	−	−	−	−	−	−	6.162	6.340	7.159	6.554
	E3-18	*Pseudalkalibacillus* sp.	−	−	−	−	−	−	−	−	−	1.107	1.288	0.578	0.991
	E5-18	*Cytobacillus* sp.	−	−	−	−	−	−	−	−	−	2.498	2.435	2.046	2.326
Las Cardas	1SR	*Erwinia* sp.	++	+++	+++	++	++	+++	+++	++++	++++	15.205	17.316	14.510	15.677
	2SR	*Pseudorhizobium* sp.	+	+	+	−	−	−	−	−	−	7.058	6.424	6.601	6.695
	4CR	*Pseudomonas* sp.	−	−	−	+++	+++	++	+++	++++	++++	14.948	14.499	14.180	14.542
Antarctic Desert	RD1	*Arthrobacter* sp.	−	−	−	−	−	−	−	−	−	6.823	7.755	7.969	7.516
	RD2	*Arthrobacter* sp.	−	−	−	−	−	−	−	−	−	6.777	8.294	10.225	8.432
	RD7	*Arthrobacter* sp.	−	−	−	−	−	−	−	−	−	9.272	8.939	7.668	8.626
	RD8	*Arthrobacter* sp.	−	−	−	−	−	−	−	−	−	9.151	9.140	8.109	8.800
	RU15	*Pseudoarthrobacter* sp.	−	−	−	−	−	−	−	−	−	10.271	10.979	9.972	10.407
	RU18	*Plantibacter* sp.	−	−	−	−	−	−	−	−	−	84.023	83.850	84.199	84.024

^*a*^ The evaluated traits included siderophore production (CAS), phosphate solubilization (PKV), nitrification (NM), and indole-3-acetic acid production. Columns I, II, and III represent triplicate measurements, while the fourth column in the IAA production assay indicates the average value (Avg.). Halo size interpretation: +, 1–2 mm; ++, 3–4 mm; +++, 5–6 mm; and ++++, 7–8 mm, measured from the edge of the bacterial colony to the edge of the halo formed.

^*b*^ −, absence.

### Co-culture assembly and short-term of relative abundance

Considering a top-down strategy, we developed a liquid co-culture of the 17 isolates, simultaneously evaluating possible interactions between the isolates, both synergistic and antagonistic, and changes in the relative abundance of the isolates. We also evaluated the influence of temperature on these parameters within an agronomically relevant range: the bacterial community was cultured independently at 15°C, 20°C, 25°C, and 30°C for two consecutive subcultures (Fig. 2; Fig. S2; Table S1).

At the beginning of the culture (t0), relative abundances of the bacterial isolates were comparable across all four temperatures. Following the first 24-h subculture (t1), marked changes in community composition were observed. Decreases in relative abundance predominated across all temperatures, affecting approximately 40% and 73% of the relative abundance of the bacterial isolates. Increases were restricted to a smaller fraction (6.7%–26.7%), and the strains that showed no variation ranged between 20% and 46.7%, with higher values observed in the culture at 25°C. By contrast, after the second subculture (t2), the community showed a stabilization pattern, with most isolates (66.7%–73.3%) maintaining their relative abundance regardless of temperature, and only minor proportions exhibiting decreases (26.7%) or increases (0%–6.7%). Notably, at t2, two isolates became predominant across temperatures: *Erwinia* sp. 1SR, which increased its relative abundance by approximately 20%–60%, and *Pseudomonas* sp. RZ5, which increased by 10%–40%, while *Plantibacter* sp. RU18 showed a consistent persistence with a moderate relative abundance across temperatures.

### Genome sequencing and phylogenomic identification of EcoBiome

To assemble the desert EcoBiome, we integrated both top-down and bottom-up design approaches, using co-culture and PGP traits of the isolates to decide. Inspired by this, we applied three selection criteria: (i) isolates must be representative of the native bacterial community of each desert, (ii) isolates must exhibit PGP traits, and (iii) isolates must show persistence or increased relative abundance across successive co-cultures. Based on these criteria, we selected one isolate from each desert: *Erwinia* sp. 1SR from Las Cardas and *Pseudomonas* sp. RZ5 from the Atacama Desert, both of which exhibited PGP traits and demonstrated the highest relative abundances during successive co-cultures. In addition, *Plantibacter* sp. RU18 from the Antarctic continent was included due to its exceptionally high production of IAA, one of the most advantageous PGP traits observed in our strain collection. This potential beneficial effect on root development positioned it as a third optimal candidate for the EcoBiome assembly, even though it did not increase in abundance in the co-culture assay but persisted throughout the experiment.

In line with continuing to investigate the metabolic capacity of the selected isolates in greater depth, we performed whole-genome sequencing. We obtained three high-quality genomes (Fig. S3), and phylogenomic trees were constructed to assign genus- and species-level taxonomy (Fig. S4). This genomic analysis allowed full taxonomic assignment for two of the three isolates: 1SR was identified as *Erwinia rhapontici* with 96% support, and RZ5 as *Pseudomonas yamanorum* with 100% support. For RU18, only the genus could be confirmed (*Plantibacter* sp.), with *Plantibacter flavus* as the closest species.

The genome assemblies (Table 2) revealed high completeness and low contamination: 99.86% completeness and 0.88% contamination for *Pseudomonas yamanorum* RZ5; 99.34% and 0.57% for *Erwinia rhapontici* 1SR; and 99.49% and 1.68% for *Plantibacter* sp. RU18. All genomes were fully circularized. In the cases of 1SR and RU18, two additional complete circular plasmids were also identified. The total assembly length was 7,257,956 bp for *Pseudomonas yamanorum* RZ5, with 6,600 predicted coding sequences (CDSs); *Erwinia rhapontici* 1SR had an assembly length of 5,439,041 bp with three contigs and 5,107 CDSs; and *Plantibacter* sp. RU18 had 4,710,248 bp and 4,463 CDSs.

**TABLE 2:** Molecular features obtained from genomic sequencing

Feature	*Pseudomonas yamanorum* RZ5	*Erwinia rhapontici* 1SR	*Plantibacter* sp. RU18
ID	RZ5	1SR	RU18
% Completeness	99.86	99.34	99.49
% Contamination	0.88	0.57	1.68
% Strain heterogeneity	9.09	0	0
*N*_50_	7,257,956	5,250,678	4,671,681
Contigs	1	3	3
% GC	60.3	54	67.4
Total length (bp)	7,257,956	5,439,041	4,710,248
Predicted genes	6,740	5,271	4,522
CDS	6,600	5,107	4,463
Pseudogenes	44	42	26
rRNA	16	22	6
tRNA	70	84	50
Genome quality	High-quality draft	High-quality draft	High-quality draft

### Genome-based prediction of secondary metabolites and metabolic pathways

To characterize the three selected strains in greater depth, we performed genomic analyses of secondary metabolites and metabolic pathways using whole-genome bioinformatic analyses.

A prediction of secondary metabolites was performed using antiSMASH (Table 3). Our results identified six different cluster types for *Erwinia rhapontici* 1SR, with two regions showing more than 50% similarity with known clusters. These corresponded to a non-ribosomal peptide synthetase (NRPS) cluster (region 1. 3) with 61% similarity to a siderophore production cluster, and region 1.2, which exhibited 94% similarity to an arylpolyene production cluster. On the other hand, *Pseudomonas yamanorum* RZ5 was predicted to have 15 gene clusters, of which three exhibited 50% or more similarity to known clusters: region 1.8 for pyocyanine (100%), region 1.10 for viscosin (50%), and region 1.12, showing 80% similarity to a pyoverdine cluster. Finally, in *Plantibacter* sp. RU18, we detected nine gene clusters, with two secondary metabolites showing similarities above 50%, corresponding to carotenoid (50%) and ε-poly-L-lysine (100%).

**TABLE 3:** Secondary metabolites identified using the antiSMASH tool from genomic sequencing of the selected isolates: *Erwinia rhapontici* 1SR, *Pseudomonas yamanorum* RZ5, and *Plantibacter* sp. RU18

	Region	Type	From	To	Most similar known cluster	Similarity
1SR	1.1	Thiopeptide	1,584,173	1,610,406	O-antigen	14%
	1.2	Arylpolyene	1,712,010	1,755,591	Aryl polyenes	94%
	1.3	NRPS	1,927,539	1,976,079	Trichrysobactin Cyclic trichrysobactin Chrysobactin Dichrysobactin	61%
	1.4	Butyrolactone	4,495,023	4,505,799	–^*a*^	–
	1.5	Redox cofactor	4,512,699	4,534,867	Lankacidin C	13%
	1.6	Betalactone	4,800,557	4,828,760	–	–
RZ5	1.1	NRPS-like	114,822	158,232	Fragin	37%
	1.2	Arylpolyene	468,077	511,652	APE Vf	35%
	1.3	RiPP-like	1,484,886	1,495,761	–	–
	1.4	NAGGN	2,272,912	2,287,788	–	–
	1.5	RiPP-like	2,588,581	2,599,432	–	–
	1.6	NI-siderophore, ectoine	3,314,421	3,344,397	–	–
	1.7	RiPP-like	4,006,034	4,018,229	–	–
	1.8	Phenazine, hserlactone	4,045,526	4,068,332	Pyocyanine	100%
	1.9	Hydrogen cyanide	4,665,090	4,681,008	Viscosin	18%
	1.10	NRPS	4,691,362	4,763,037	Viscosin	50%
	1.11	Betalactone	4,829,190	4,853,062	Fengycin	13%
	1.12	NRP-metallophore, NRPS	4,931,025	5,013,553	Pyoverdine SMX-1	80%
	1.13	NRPS	5,079,194	5,132,138	Pf-5 pyoverdine	17%
	1.14	Redox cofactor	6,667,768	6,689,924	Lankacidin C	13%
	1.15	RiPP-like	7,207,429	7,218,274	–	–
RU18	1.1	NI-siderophore	756,238	785,833	FW0622	25%
	1.2	Terpene	1,176,479	1,197,432	Carotenoid	50%
	1.3	T3PKS	1,704,021	1,745,214	5-acetyl-5,10-dihydrophenazine-1-carboxylic acid 5-(2-hydroxyacetyl)-5,10-dihydrophenazine-1-carboxylic acid Endophenazine A1 Endophenazine F Endophenazine G	17%
	1.4	NAPAA	1,877,633	1,911,640	ε-Poly-L-lysine	100%
	1.5	Betalactone	2,230,941	2,258,353	Pentalenolactone	15%
	1.6	T3PKS	2,646,843	2,687,961	–	–
	1.7	Hydrogen cyanide	3,419,832	3,432,890	Aborycin	21%
	1.8	Proteusin, RiPP-like	3,479,322	3,499,606	–	–
	1.9	Arylpolyene	3,847,807	3,888,823	–	–

^*a*^ −, absence.

Additionally, we performed a genomic analysis using KofamKOALA to assign KEGG Orthologs (Table S2). Two genes (*nirBD* and *nirD*) related to the dissimilatory nitrate reduction pathway were identified in *Plantibacter* sp. RU18. Furthermore, *Erwinia rhapontici* 1SR exhibited all screened genes associated with the phosphate uptake pathway (*pstS*, *pstA*, *pstC*, *pstB*, *phoA, phoR*, *phoU,* and *phoB*), the iron uptake pathway (*fhuB*, *fhuC*, *fhuD*, *feoA,* and *fur*), and the siderophore pathway (*fepB*, *fepC*, *fepD*, and *fepG*). In contrast, these genes were only partially present in *Pseudomonas yamanorum* RZ5 and *Plantibacter* sp. RU18.

All isolates possessed the *efeB* gene, involved in the iron transport pathway, as well as some genes related to the IAA synthesis pathway (*mao, trpA*, *trpB*, *trpC,* and *trpS*). Additionally, ACC deaminase (*acdS*) was absent only in *Erwinia rhapontici* 1SR.

We also analyzed metabolic pathways associated with biofilm formation, identifying 20 genes. The results indicated that *Pseudomonas yamanorum* RZ5 harbored all biofilm-related genes, while *Erwinia rhapontici* 1SR contained 13 genes, and *Plantibacter* sp. RU18 had 3 genes. Furthermore, all isolates presented the *murJ* gene, associated with flagellar activity.

Finally, we analyzed genes associated with tolerance to abiotic stress conditions. In all three isolates, we detected genes related to heat stress tolerance (*smpB*, *dnaJ*, *dnaK*, *grpE*, *groES*, *clpB*, *clpX*, and *htpX*) and genes associated with tolerance to drought and saline conditions (*nhaA*, *proB*, *proS*, *kdpA*, *kdpB*, *kdpF,* and *kdoC*).

### Growth kinetics and competitive interactions in the EcoBiome

To assess the effect of temperature on the growth kinetics of the selected isolates, growth curves were generated (Fig. S5). The maximal optical density (OD_max_) and the doubling time (DT) were calculated (Table 4). The results indicated that the optimal temperature range for achieving the highest OD_max_ was between 15°C and 25°C. Additionally, a pronounced decrease in OD_max_ was observed for *Plantibacter* sp. RU18 at 30°C, whereas *Pseudomonas yamanorum* RZ5 and *Erwinia rhapontici* 1SR exhibited only slight decreases. Regarding the DT values, the lowest DT was recorded at 30°C. The second lowest DT occurred at 20°C, representing a balance between higher proliferation rates (lower DT) and higher OD_max_ values for all three isolates, followed by 25°C and 15°C.

**TABLE 4:** Bacterial growth kinetics at different temperatures in selected isolates^*a*^

Isolate	15°C		20°C		25°C		30°C	
	OD_max_	DT (h)	OD_max_	DT (h)	OD_max_	DT (h)	OD_max_	DT (h)
RZ5	1.60	10.26	1.55	7.67	1.67	9.06	1.44	3.55
1SR	1.38	10.81	1.45	7.93	1.30	8.52	1.15	4.42
RU18	1.28	16.72	1.28	10.83	1.28	11.44	0.39	7.85

^*a*^ OD_max_ and DT were calculated based on each growth curve.

Additionally, proliferation dynamics were evaluated at 15°C, 20°C, 25°C, and 30°C to assess bacterial competition or synergism in both pairwise and three-isolate (EcoBiome) cultures (Fig. S6). The results showed no evidence of competition (inhibition) or attraction at any evaluated temperature, but rather complementary growth among the different combinations.

### Functional characterization of plant growth-promoting traits and abiotic stress tolerance in the EcoBiome

**Fig 3:**
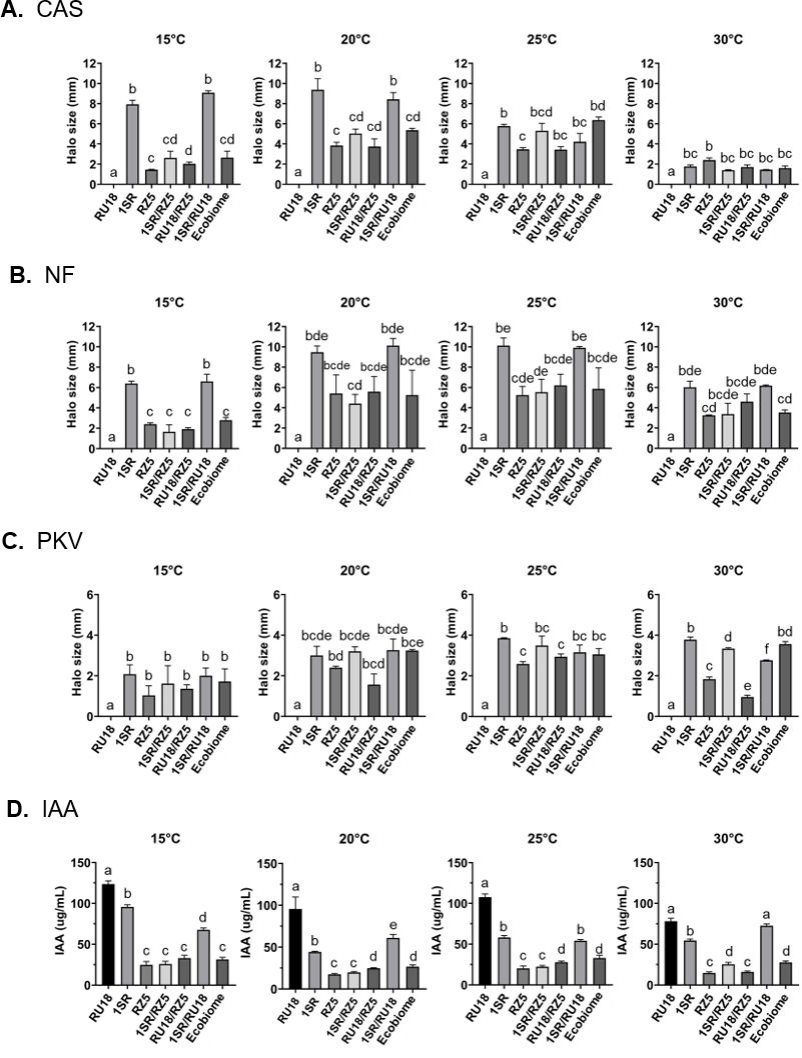
Characterization of plant growth-promoting traits in *Erwinia rhapontici* 1SR, *Pseudomonas yamanorum* RZ5, and *Plantibacter* sp. RU18, evaluated individually, in pairwise combinations, and as the EcoBiome at four incubation temperatures. The *in vitro* assays included (**A**) siderophore production on CAS agar, (**B**) nitrification process on NM agar, (**C**) phosphate solubilization on PKV agar, and (**D**) IAA production with Salkowski reagent.

To evaluate the contribution of each isolate to PGP traits within the EcoBiome, a characterization assay was performed at four incubation temperatures (15°C, 20°C, 25°C, and 30°C) using individual isolates, in pairwise combinations, and the EcoBiome (Fig. 3; Fig. S7).

The results revealed differences in siderophore production (CAS), nitrification (NM), phosphate solubilization (PKV), and IAA production depending on the incubation temperature. Specifically, the highest siderophore production was observed at 20°C by *Erwinia rhapontici* 1SR, both individually and in combination with *Plantibacter* sp. RU18. In contrast, the EcoBiome showed no significant differences compared to *Pseudomonas yamanorum* RZ5 alone or its combinations with *Plantibacter* sp. RU18 or *Erwinia rhapontici* 1SR. A similar trend was observed at 15°C and 25°C, although with a smaller halo size in all cases. At 30°C, the isolates, pairwise combinations, and the EcoBiome exhibited the lowest siderophore production, with no significant differences among them.

Regarding nitrification and phosphate solubilization values, the highest values were observed at 20°C and 25°C, with no significant differences between EcoBiome, *Erwinia rhapontici* 1SR, *Pseudomonas yamanorum* RZ5, and their combinations with *Plantibacter* sp. RU18. These values decreased at both 30°C and 15°C.

IAA production results indicated that *Plantibacter* sp. RU18 was the highest producer at all four tested temperatures, showing significant differences compared to the other isolates and their combinations. Meanwhile, the EcoBiome exhibited its highest IAA production at 25°C, with no significant differences compared to the combination of *Plantibacter* sp. RU18 and *Pseudomonas yamanorum* RZ5.

**Fig 4:**
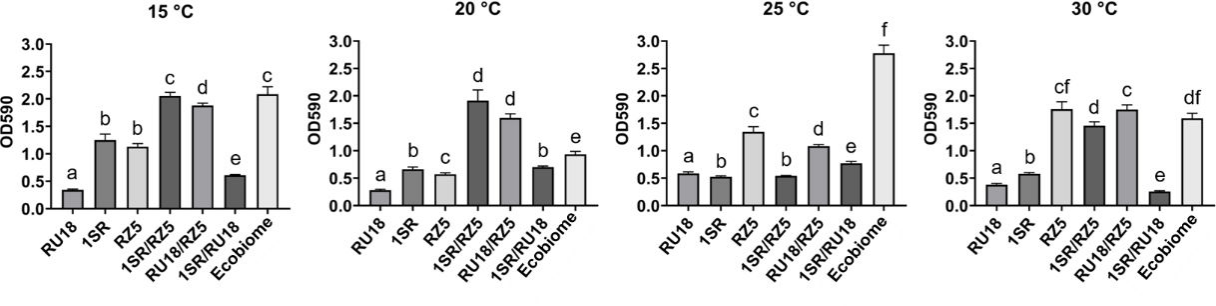
Biofilm formation assay of the EcoBiome and isolates. The assay was performed with *Erwinia rhapontici* 1SR, *Pseudomonas yamanorum* RZ5, and *Plantibacter* sp. RU18 individually, in pairwise combinations, and as the EcoBiome at four incubation temperatures.

Additionally, two tests were performed to assess the adherence capacity and establishment of the EcoBiome: a biofilm formation assay (Fig. 4) and an assay evaluating proliferation under water deficit using polyethylene glycol (PEG) in the culture medium (Fig. S8). The results showed that the EcoBiome formed significantly more biofilm at 25°C compared to each isolate or pairwise combination. At 15°C, 20°C, and 30°C, biofilm formation was lower, but the differences were not statistically significant compared to the bacterial pairs at each temperature.

On the other hand, *in vitro* tolerance to water deficit revealed that all isolates tolerate both PEG 5% and PEG 10% conditions relative to the control (PEG 0%). The EcoBiome exhibited greater tolerance (measured as higher proliferation) at 25°C, followed by 20°C, 30°C, and 15°C for both PEG concentrations.

### Influence of EcoBiome on native soil bacterial communities under different soil moisture conditions

**Fig 5:**
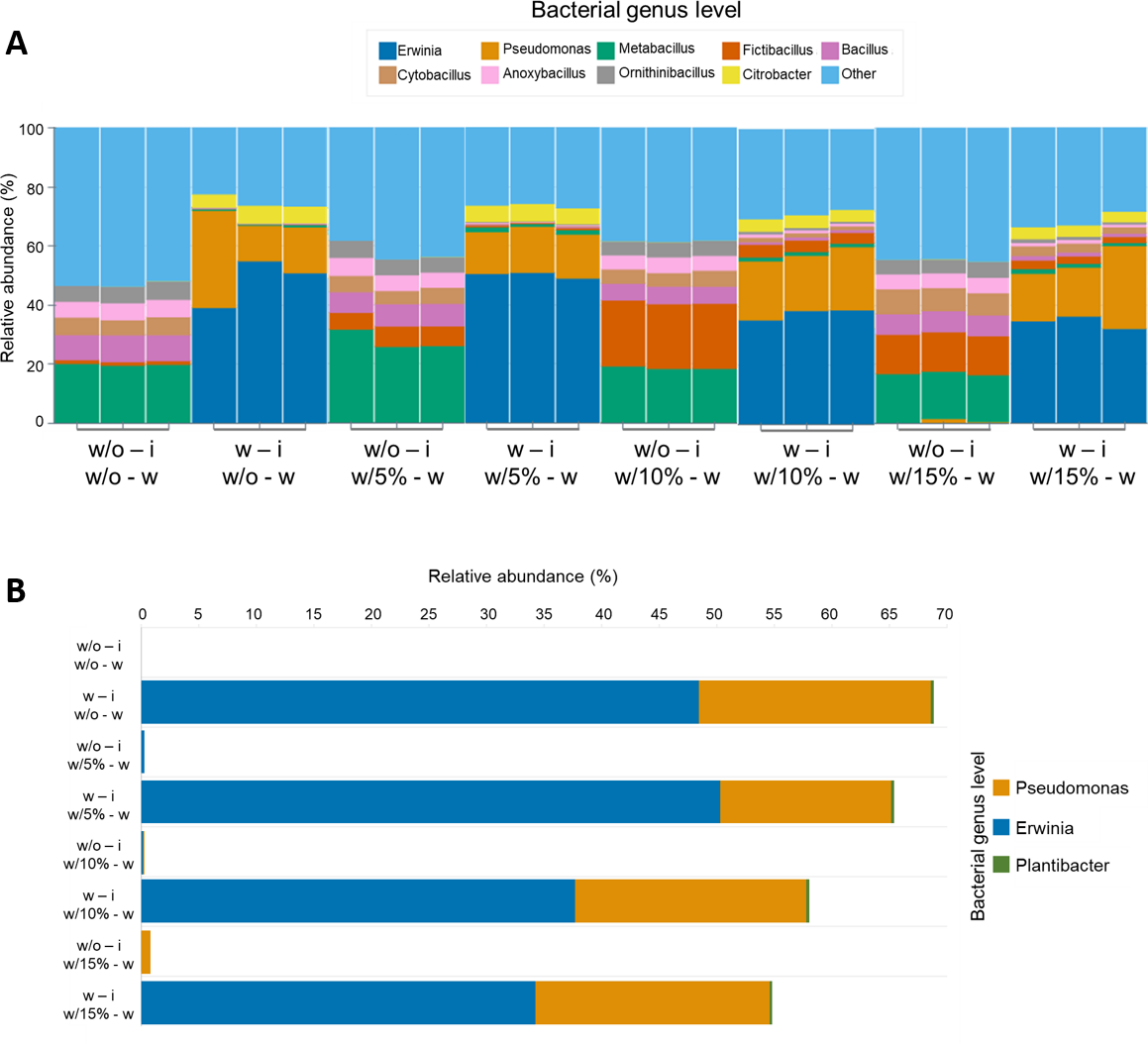
Relative abundance of soil samples in different treatments. (**A**) Total taxonomic composition at the bacterial genus level and their respective treatments. (**B**) Specific relative abundance of EcoBiome as a bacterial inoculum in a desert soil sample. Without inoculum (w – i), with inoculum (w – i), without water (w/o – w), with 5% soil moisture (w/5% – w), with 10% soil moisture (w/10% – w), and with 15% soil moisture (w/15% – w).

To determine the influence of EcoBiome inoculation and its establishment, we used desert soil and analyzed the changes in the native bacterial community at the genus level before and after inoculation. We also assessed how the community responded to different soil moisture conditions (5%, 10%, and 15%) in both inoculated and non-inoculated treatments (Fig. 5).

The bacterial composition at the genus level showed variations in response to the evaluated treatments (presence/absence of inoculation and different soil moisture) (Fig. 5A). Overall, bacterial communities were dominated by *Metabacillus* and *Bacillus* in non-inoculated treatments (w/o – i), whereas *Erwinia* and *Pseudomonas* predominated in inoculated soils (w – i).

Regarding the different soil moisture treatments (5%, 10%, and 15%), non-inoculated treatments (w/o – i and w/5% – w) exhibited higher proportions of *Metabacillus* and *Fictibacillus*, along with a slight decrease in *Bacillus*, while inoculated treatments (w – i and w/5% – w) were dominated by *Erwinia*, with a relevant contribution of *Pseudomonas*.

At 10% soil moisture, a greater dominance of *Fictibacillus* was observed, accompanied by a slight decrease in *Metabacillus* and an increase in the relative abundance of *Bacillus* and *Cytobacillus*. In contrast, in the inoculated treatment, *Erwinia* and *Pseudomonas* remained the predominant genera, although with a relative reduction compared to the 5% moisture treatment or the absence of moisture treatment.

Finally, at 15% soil moisture, the bacterial community showed a pattern similar to that observed at 10%, with a further increase in the relative abundance of *Bacillus*, *Cytobacillus*, and *Anoxybacillus*, particularly in non-inoculated treatments, whereas in inoculated treatments, *Erwinia* and *Pseudomonas* maintained their dominance.

Regarding the relative abundance of bacterial genera that comprise the EcoBiome (Fig. 5B), our results show a differential response to soil inoculation and soil moisture gradient. In the absence of inoculation (w/o – i), bacterial genera associated with EcoBiome were virtually absent, except at 5%, 10%, and 15% soil moisture, where only a low relative abundance of the genera *Pseudomonas* and *Erwinia* was detected. In contrast, inoculation with EcoBiome in the soil (w – i) promoted an increase in the relative abundance of the *Erwinia* and *Pseudomonas* genera; however, *Erwinia* exhibited a higher relative abundance in soils without moisture or with 5% moisture. In contrast, the *Plantibacter* genus showed a low but constant relative abundance in all treatments with inoculation.

## DISCUSSION

In this study, we combined top-down and bottom-up design strategies to construct an EcoBiome derived from three desert environments. The top-down component enabled an initial reduction of microbial complexity by selecting bacterial isolates capable of coexisting under laboratory conditions, while the bottom-up approach incorporated detailed functional and genomic characterization to identify complementary PGP traits. Integrating these two perspectives allowed us to assemble a minimal but functionally robust consortium that reflects ecological attributes of native desert communities while remaining experimentally tractable.

The liquid co-culture assay of the 17 isolates served as a first ecological filter, revealing taxa that persisted or increased in relative abundance under agronomically relevant temperatures. *Erwinia rhapontici* 1SR and *Pseudomonas yamanorum* RZ5 showed the highest increases during successive subcultures, suggesting competitive advantages and compatibility within a mixed community. Although *Plantibacter* sp. RU18 did not increase in abundance, its consistent persistence and distinctive metabolic profile, particularly its exceptional IAA production, supported its inclusion in the EcoBiome. Collectively, these three isolates represent phylogenetically diverse bacterial groups originating from warm and cold desert soils, capturing ecological and functional variability across extreme environments.

PGP trait analysis revealed complementary capabilities among isolates. *Erwinia rhapontici* 1SR was the only strain displaying all four evaluated traits (siderophore production, phosphate solubilization, nitrification, and IAA synthesis), consistent with previous reports of PGP activity in non-pathogenic *Erwinia* species associated with crops (73, 74). *Pseudomonas yamanorum* RZ5 exhibited multiple PGP functions, in line with the broad literature describing *Pseudomonas* spp. as versatile plant-beneficial bacteria with strong colonization and metabolic capacities (6, 23, 75, 76). *Plantibacter* sp. RU18 stood out as the highest IAA producer in the entire collection, a trait of particular relevance for root development and plant–microbe interactions. This observation aligns with previous findings in *Plantibacter flavus*, where auxin and cytokinin biosynthesis contributed to enhanced growth of *Arabidopsis thaliana* (77).

Genome mining further supported the functional profiles of the isolates. Both *E. rhapontici* 1SR and *P. yamanorum* RZ5 carried siderophore-related biosynthetic gene clusters, confirming the potential for iron acquisition mechanisms that were also evident in halo formation assays. In contrast, the presence of nitrogen and phosphate-related genes in *Plantibacter* sp. RU18 was not reflected in detectable phenotypes, emphasizing the importance of combining genomic and culture-based assessments to accurately evaluate microbial functions. Additional genomic features, including phosphate uptake systems in *E. rhapontici* 1SR and biofilm-associated genes in *P. yamanorum* RZ5, further highlighted complementary ecological roles within the consortium. Previous reports have demonstrated that biochemical assays combined with bioinformatics analyses based on whole-genome information constitute a complementary approach that not only provides more comprehensive insights but also enables deeper and more robust outcomes, including the identification of novel biosynthetic pathways, reconstruction of metabolic routes, and the discovery of metabolites with an emphasis on biosynthetic processes and potential biotechnological applications derived from microorganisms (75, 78).

The secondary metabolite repertoire provided insights into additional ecological strategies. *P. yamanorum* RZ5 contained gene clusters for pyocyanin and pyoverdine, both associated with iron metabolism and biofilm formation (79–82). These traits may enhance survival in iron-limited desert soils and promote colonization in plant-associated niches. *Erwinia rhapontici* 1SR encoded arylpolyenes, pigments associated with protection against photo-oxidative stress (83), and siderophore clusters with similarities to chrysobactin systems, indicating multiple strategies for coping with UV radiation and nutrient scarcity (84). *Plantibacter* sp. RU18 harbored an ε-poly-L-lysine biosynthetic cluster, which may provide antimicrobial defense, although this activity was not evident in the pairwise antagonism assays.

The ability to form stable multispecies biofilms is a critical attribute for niche colonization and collective stress protection (85). The EcoBiome exhibited significantly higher biofilm production at 25°C compared with individual isolates and pairwise combinations, consistent with reports showing enhanced biofilm formation in microbial consortia (6). Since biofilms mediate physical protection, nutrient exchange, and microhabitat stabilization that protect microorganisms and enhance their survival (86), their formation may support the establishment and performance of the EcoBiome in soil environments. Interaction assays further indicated the absence of antagonism among isolates, supporting their compatibility in a microbial inoculant.

Specifically, the assay used to determine metal acquisition activity (siderophore production) revealed an effect attributable to incubation temperature. For example, isolate 1SR exhibited its highest replication rate at 15°C and 20°C (Fig. S5), temperatures at which it also showed greater siderophore production both individually and in paired cultures. In contrast, at 30°C, all isolates displayed reduced replication rates and, consequently, lower metabolic activity, reflected in decreased siderophore production under this condition across all treatments. In this context, siderophores, as non-essential secondary metabolites, are typically produced toward the end of the exponential growth phase, and their release into the environment depends on microbial ecological physiology and energetic trade-offs (87, 88).

On the other hand, assays associated with nitrification and inorganic phosphate solubilization (PKV) did not show significant differences between the EcoBiome treatment and the paired combinations and/or individual isolates at any of the temperatures evaluated. However, higher specific metabolic activity was observed in assays conducted at 20°C and 25°C. These findings suggest that bacterial metabolic activity may exhibit a temperature-dependent response. This pattern has been reported by Adekanmbi et al. (89), who demonstrated that intracellular metabolic processes in soil microorganisms display marked thermal sensitivity, and Seidel et al. (90), who showed that temperature shifts can alter metabolic activity in sediments by modifying transcriptional regulation.

Finally, the assay related to IAA production showed a decrease in detected levels in the EcoBiome treatment across all four temperatures evaluated. In contrast, the highest production levels were observed in isolate RU18, followed by its combination with isolate 1SR. We attribute this pattern to resource competition, considering that IAA biosynthesis relies on tryptophan as its primary precursor. Therefore, in oligotrophic soils such as the Atacama Desert, competition for amino acids is likely intense. In contrast, under mesotrophic or eutrophic conditions, such competition may be reduced, as nutrients, including tryptophan, are not typically limiting. Supporting this interpretation, Zhang et al. (91) reported, through large-scale genomic analyses, that approximately 82% of the bacterial genomes examined contained pathways associated with IAA biosynthesis, suggesting that competition for this metabolic trait may represent a significant ecological interaction in soil environments.

Additionally, these effects may be explained by cell density-dependent regulatory mechanisms in the environment, such as quorum-sensing (QS) processes, which can modulate the expression of metabolically costly traits. In this context, QS systems have been shown to regulate IAA biosynthesis (92), siderophore production (93), and nitrogen (94) and phosphorus transformation pathways (95).

Overall, the selection and functional characterization of *Erwinia rhapontici* 1SR, *Pseudomonas yamanorum* RZ5, and *Plantibacter* sp. RU18 revealed a complementary set of traits related to nutrient mobilization, hormone production, iron acquisition, stress tolerance, and biofilm formation. The absence of antagonistic interactions and the capacity to withstand water-deficit conditions underscore the stability and ecological coherence of the EcoBiome. These observations have been described by Sharma et al. (96), highlighting how combining the metabolic functions of bacterial cultures through the use of consortia can improve plant growth.

These findings position the EcoBiome as a promising microbial resource for biotechnological applications aimed at enhancing plant resilience and productivity in arid and semi-arid agroecosystems. Future work should validate its performance under greenhouse and field conditions and assess its interactions with plant hosts and soil microbiomes.

### Conclusion

In this study, we developed a desert-derived microbial EcoBiome by integrating ecological filtering and functional characterization. The combination of top-down and bottom-up strategies allowed us to identify three complementary bacteria: *Erwinia rhapontici* 1SR, *Pseudomonas yamanorum* RZ5, and *Plantibacter* sp. RU18, which collectively expressed key plant growth-promoting traits, including nutrient mobilization, siderophore and auxin production, biofilm formation, and tolerance to drought-related stress.

The EcoBiome demonstrated functional complementarity and stability, with no antagonistic interactions among isolates, and showed enhanced biofilm formation and resilience under reduced water availability. These characteristics highlight its potential to establish itself in the soil, which we have verified through inoculation in desert soils with different moisture contents, observing successful establishment in all treatments performed.

Overall, our findings provide a framework for the rational design of simplified microbial EcoBiome inspired by desert environments and identify a promising three-member inoculant for further evaluation. Future greenhouse and field studies will be essential to validate its effectiveness in improving plant growth, soil function, and crop productivity under environmental stress.

## SUPPLEMENTAL MATERIAL

Figure S1 aem.00103-26-s0001.tif Schematic representation of the bacterial co-culture method at different temperatures and DNA isolation time points.

Figure S2 aem.00103-26-s0002.tif Relative abundance dynamics of bacterial isolates grown at different temperatures.

Figure S3 aem.00103-26-s0003.tif Circular representation of genomic features of bacterial isolates.

Figure S4 aem.00103-26-s0004.tif Phylogenomic tree of the EcoBiome strains and related taxa based on whole genome comparisons.

Figure S5 aem.00103-26-s0005.tif Growth curves of bacterial isolates under different experimental conditions.

Figure S6 aem.00103-26-s0006.tif Representative agar plate assays showing growth and interaction patterns of bacterial isolates under different temperature conditions.

Figure S7 aem.00103-26-s0007.tif Biochemical assays of plant growth-promoting traits.

Figure S8 aem.00103-26-s0008.tif Water stress tolerance assay with different polyethylene glycol concentrations.

Tables S1 and S2 aem.00103-26-s0009.docx Table S1, changes in relative abundance in co-culture; Table S2, search for genes related to PGP traits.

Graphical abstract aem.00103-26-s0010.tiff Visual depiction of the study.

ASM does not own the copyrights to Supplemental Material that may be linked to, or accessed through, an article. The authors have granted ASM a non-exclusive, world-wide license to publish the Supplemental Material files. Please contact the corresponding author directly for reuse.
