# Supplementary figures and images for: Development of a plant growth-promoting bacterial EcoBiome derived from desert soil isolates
Source: Appl Environ Microbiol. 2026 Apr 13;92(5):e00103-26. doi: 10.1128/aem.00103-26 (PMC13188922; doi:10.1128/aem.00103-26)

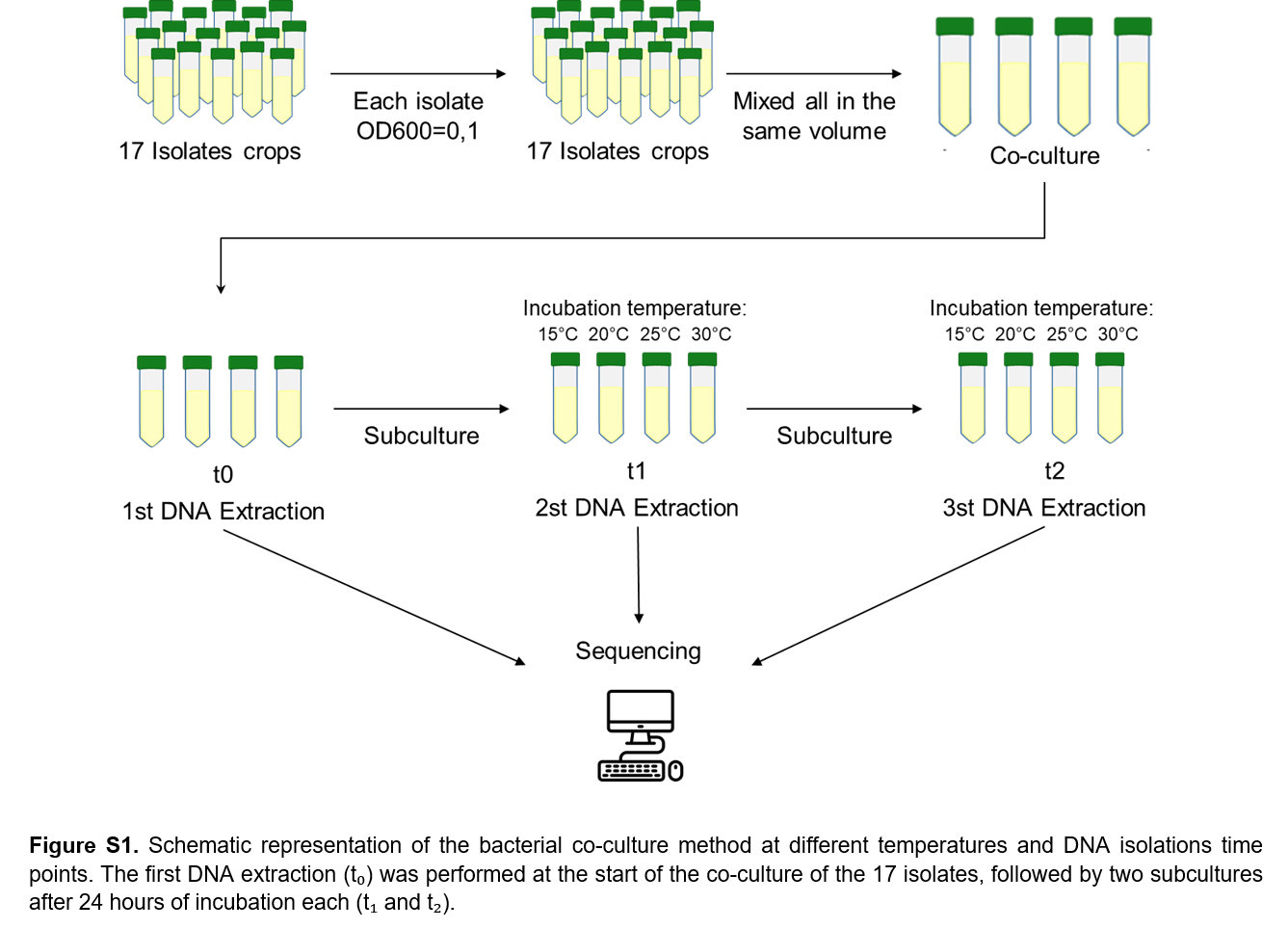

Supplement: Figure S1 — Schematic representation of the bacterial co-culture method at different temperatures and DNA isolation time points. [file aem.00103-26-s0001.tif]

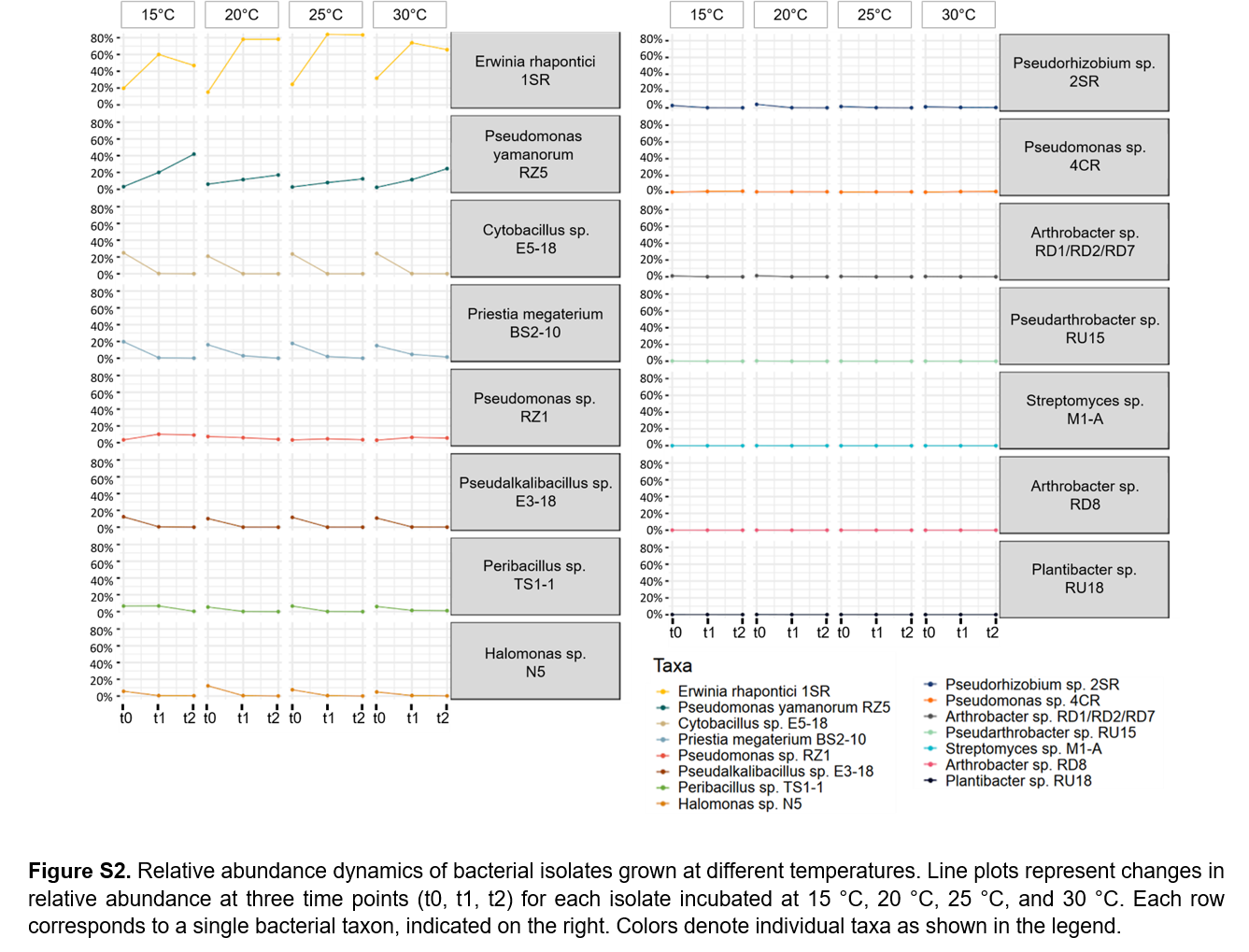

Supplement: Figure S2 — Relative abundance dynamics of bacterial isolates grown at different temperatures. [file aem.00103-26-s0002.tif]

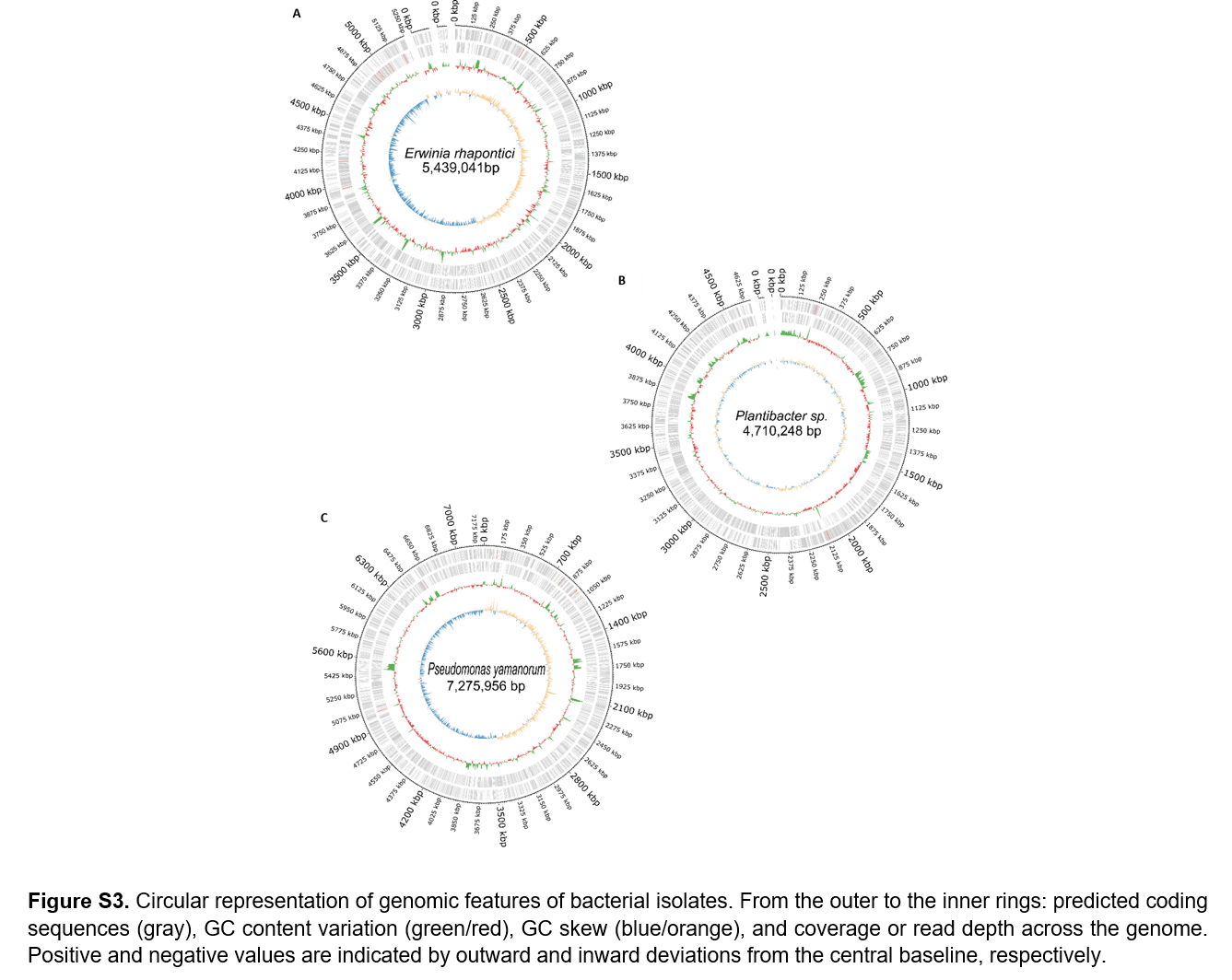

Supplement: Figure S3 — Circular representation of genomic features of bacterial isolates. [file aem.00103-26-s0003.tif]

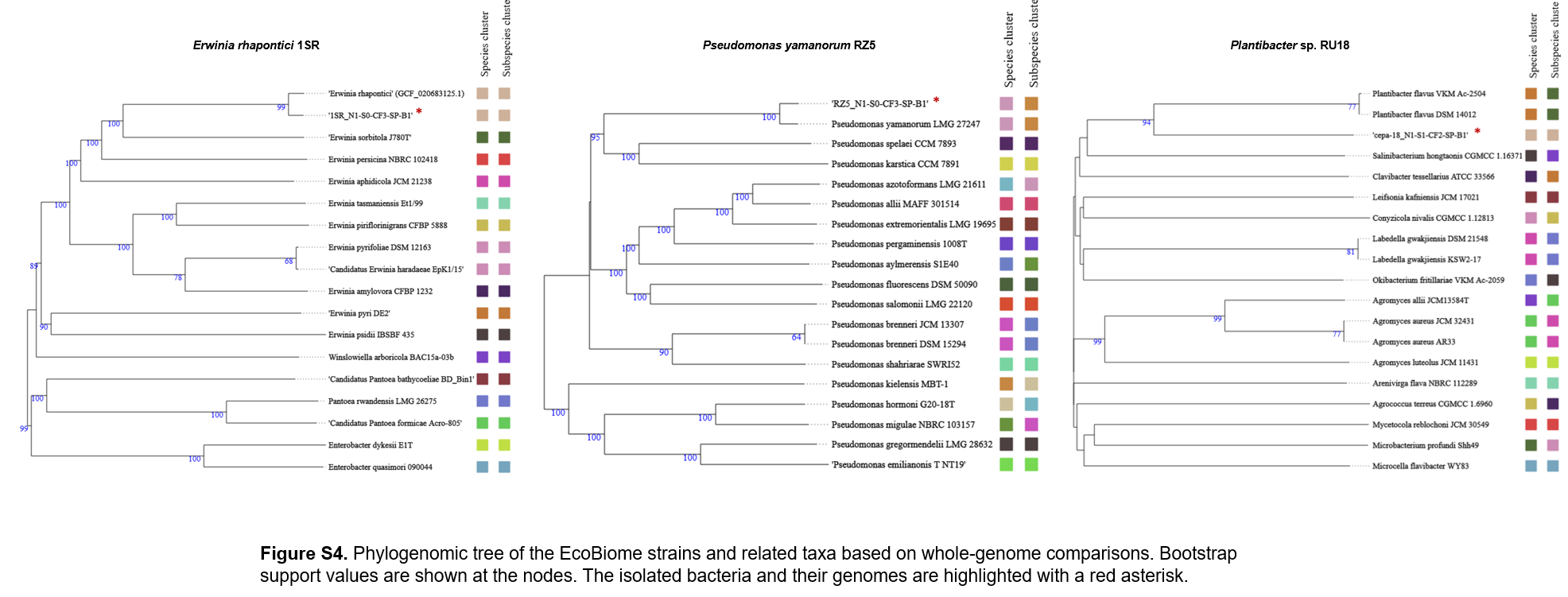

Supplement: Figure S4 — Phylogenomic tree of the EcoBiome strains and related taxa based on whole genome comparisons. [file aem.00103-26-s0004.tif]

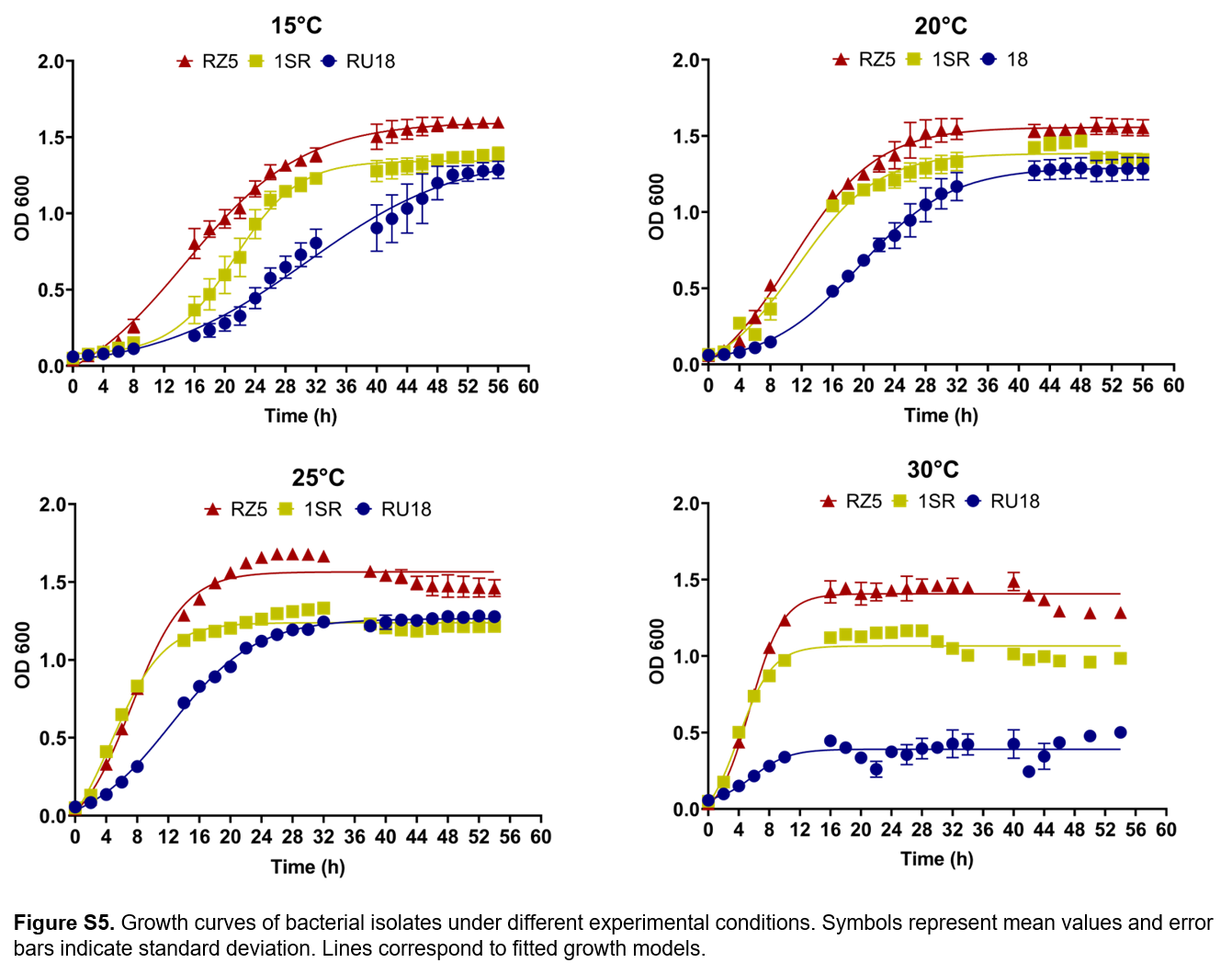

Supplement: Figure S5 — Growth curves of bacterial isolates under different experimental conditions. [file aem.00103-26-s0005.tif]

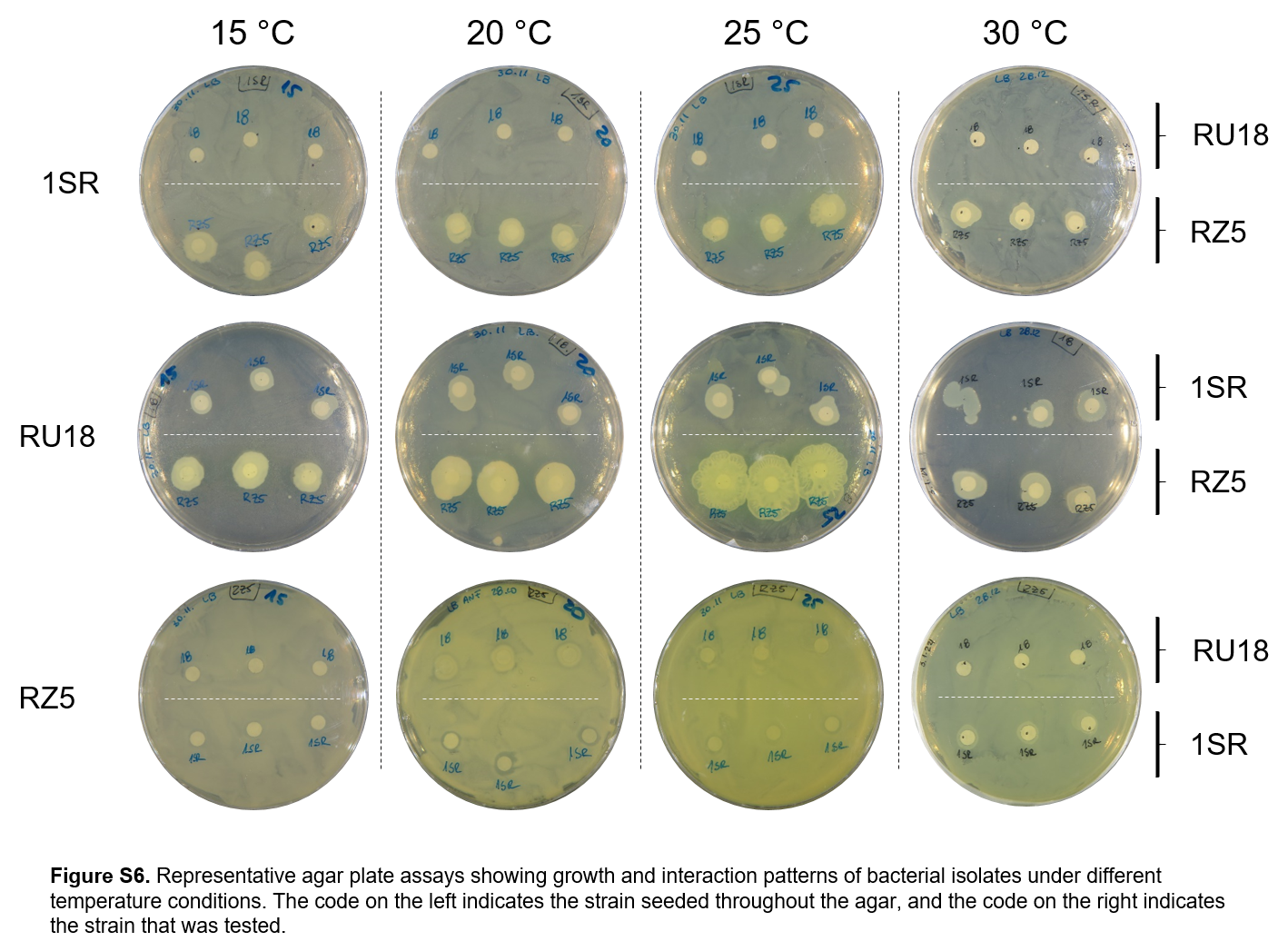

Supplement: Figure S6 — Representative agar plate assays showing growth and interaction patterns of bacterial isolates under different temperature conditions. [file aem.00103-26-s0006.tif]

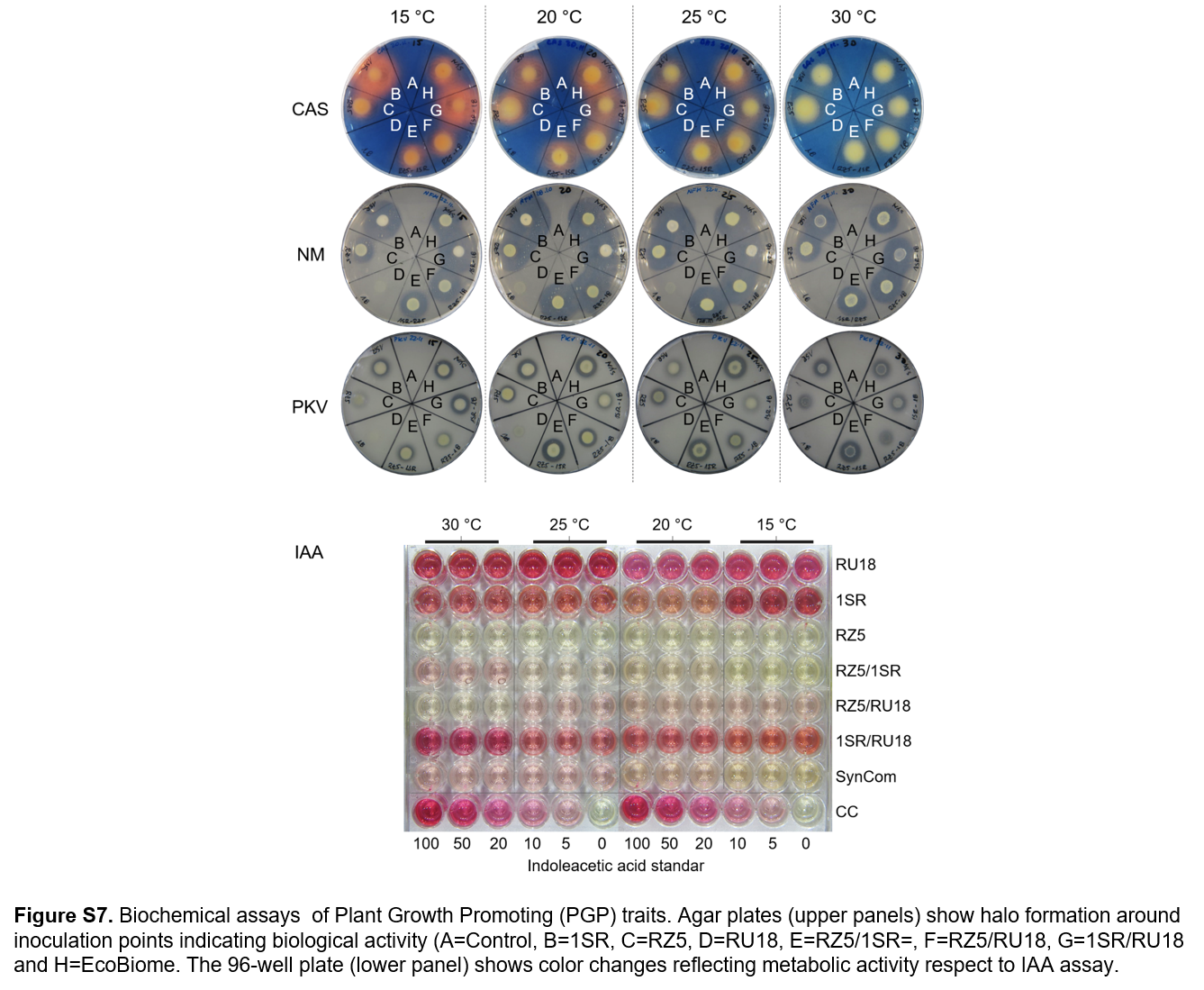

Supplement: Figure S7 — Biochemical assays of plant growth-promoting traits. [file aem.00103-26-s0007.tif]

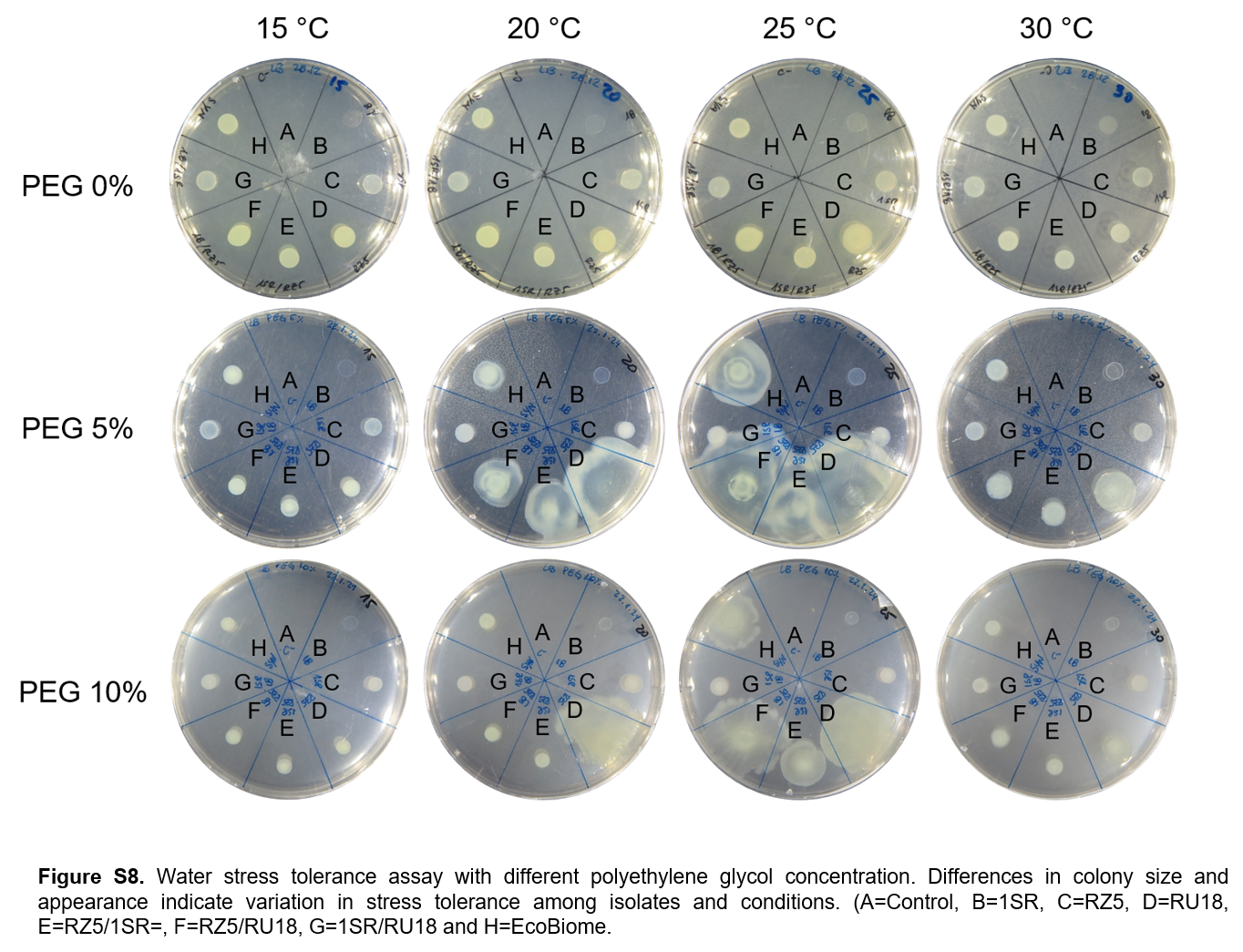

Supplement: Figure S8 — Water stress tolerance assay with different polyethylene glycol concentrations. [file aem.00103-26-s0008.tif]

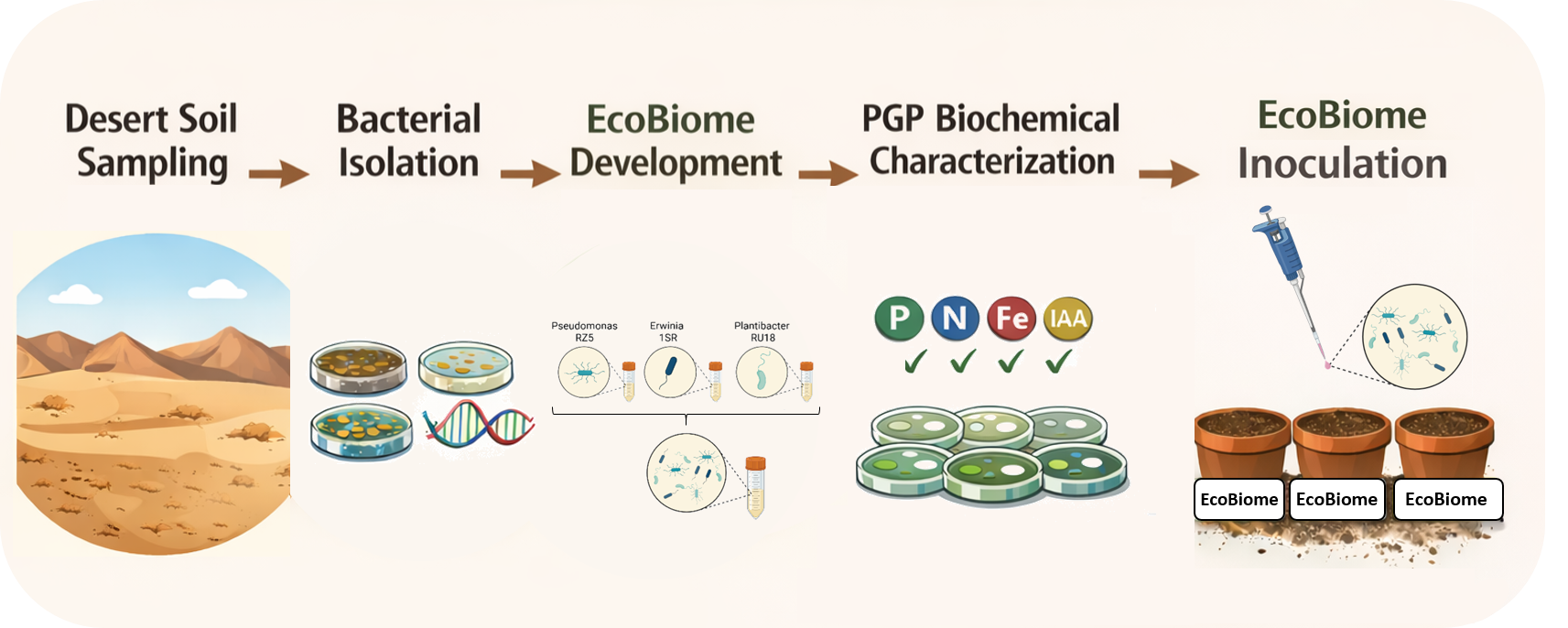

Supplement: Graphical abstract — Visual depiction of the study. [file aem.00103-26-s0010.tiff]
