# Supplementary material for: Development of a plant growth-promoting bacterial EcoBiome derived from desert soil isolates
Source: Appl Environ Microbiol. 2026 Apr 13;92(5):e00103-26. doi: 10.1128/aem.00103-26 (PMC13188922; doi:10.1128/aem.00103-26)
Supplement: Tables S1 and S2 — Table S1, changes in relative abundance in co-culture; Table S2, search for genes related to PGP traits. [file aem.00103-26-s0009.docx]

| **Table S1.** Percentage of bacterial taxa showing increases, decreases, or no change in relative abundance across four culture temperatures, evaluated at two experimental time points. | | | | | | | | |
| --- | --- | --- | --- | --- | --- | --- | --- | --- |
|  |  |  |  |  |  |  |  |  |
|  | **15°C** | | **20°C** | | **25°C** | | **30°C** | |
|  | t1 | t2 | t1 | t2 | t1 | t2 | t1 | t2 |
| Increases | 26,7% | 6,7% | 6,7% | 0% | 13,3% | 0% | 26,7% | 6,7% |
| Decreases | 46,7% | 26,7% | 73,3% | 26,7% | 40% | 26,7% | 53,3% | 26,7% |
| Unchanged | 26,7% | 66,7% | 20% | 73,3% | 46,7% | 73,3% | 20% | 66,7% |

| **Table S2.** Genomic screening for metabolic pathways associated with PGP traits for three isolates: *Erwinia rhapontici* 1SR, *Pseudomona yamanorum* RZ5, and *Plantibacter* sp. RU18. The KEGG Orthologs (KO) codes of the genes present in the metabolic pathways are provided, and the last three columns indicate the number of gene copies in each genome. | | | | | | |
| --- | --- | --- | --- | --- | --- | --- |
| *Genes involved to nitrogen, phosphorus, and iron metabolism.* | | | |  |  |  |
| **Metabolic Traits** | **Annotation Entry (KO)** | **Gene** | **Product Name** | **1SR** | **RZ5** | **RU18** |
| Dissimilatory nitrate reduction | K00362 | *nirB* | Nitrite reductase (NADH) large subunit | 2 | 0 | 1 |
|  | K00363 | *nirD* | Nitrite reductase (NADH) small subunit | 0 | 0 | 1 |
| Phosphate uptake | K02040 | *pstS* | Phosphate transport system substrate-binding protein | 2 | 4 | 1 |
|  | K02038 | *pstA* | Phosphate transport system permease protein | 2 | 2 | 1 |
|  | K02037 | *pstC* | Phosphate transport system permease protein | 2 | 2 | 1 |
|  | K02036 | *pstB* | Phosphate transport system ATP-binding protein | 2 | 2 | 1 |
|  | K01077 | *phoA* | Alkaline phosphatase | 1 | 0 | 0 |
|  | K07636 | *phoR* | Phosphate regulon sensor histidine kinase PhoR | 1 | 1 | 0 |
|  | K02039 | *phoU* | Phosphate transport system protein | 1 | 1 | 1 |
|  | K07657 | *phoB* | Phosphate regulon response regulator PhoB | 1 | 1 | 0 |
| Iron Uptake | K02015 | *fhuB* | Iron complex transport system permease protein | 2 | 3 | 1 |
|  | K10829 | *fhuC* | Ferric hydroxamate transport system ATP-binding protein | 1 | 0 | 1 |
|  | K02016 | *fhuD* | Iron complex transport system substrate-binding protein | 4 | 3 | 0 |
|  | K04758 | *feoA* | Ferrous iron transport protein A | 1 | 0 | 0 |
|  | K03711 | *Fur* | Fur family transcriptional regulator, ferric uptake regulator | 1 | 1 | 1 |
| Iron (II) transport | K16301 | *efeB* | Deferrochelatase/peroxidase EfeB | 1 | 1 | 1 |
| Siderophore | K23185 | *fepB* | Ferric enterobactin transport system substrate-binding protein | 1 | 0 | 1 |
|  | K23186 | *fepD* | Iron-siderophore transport system permease protein | 1 | 0 | 6 |
|  | K23187 | *fepG* | Iron-siderophore transport system permease protein | 0 | 0 | 6 |
|  | K23188 | *fepC* | Iron-siderophore transport system ATP-binding protein | 1 | 0 | 3 |
|  |  |  |  |  |  |  |
| *Genes related to phytohormone production* | | | |  |  |  |
| **Metabolic Traits** | **Annotation Entry (KO)** | **Gene** | **Product Name** | **1SR** | **RZ5** | **RU18** |
| IAA synthesis | K00274 | *mao* | Monoamine oxidase | 0 | 0 | 1 |
|  | K01695 | *trpA* | Tryptophan synthase alpha chain [EC:4.2.1.20] | 1 | 1 | 1 |
|  | K01696 | *trpB* | Tryptophan synthase subunit beta | 1 | 1 | 1 |
|  | K01609 | *trpC* | Indole-3-glycerol phosphate synthase TrpC | 0 | 1 | 0 |
|  | K01867 | *trpS* | Tryptophan–tRNA ligase | 1 | 1 | 1 |
| ACC deaminase | K01505 | *acdS* | ACC deaminase | 0 | 1 | 1 |
|  |  |  |  |  |  |  |
| *Genes associated with stress tolerance.* | | | |  |  |  |
| **Metabolic Traits** | **Annotation Entry (KO)** | **Gene** | **Product Name** | **1SR** | **RZ5** | **RU18** |
| Heat shock protein | K03664 | *smpB* | SsrA-binding protein SmpB | 1 | 1 | 1 |
|  | K03686 | *dnaJ* | Molecular chaperone DnaJ | 1 | 1 | 1 |
|  | K04043 | *dnaK* | Molecular chaperone DnaK | 1 | 1 | 1 |
|  | K03687 | *grpE* | Nucleotide exchange factor GrpE | 1 | 1 | 1 |
|  | K04078 | *groES* | Chaperonin GroES | 1 | 1 | 1 |
|  | K03695 | *clpB* | ATP-dependent chaperone ClpB | 1 | 1 | 1 |
|  | K03544 | *clpX* | ATP-dependent Clp protease ATP-binding subunit ClpX | 2 | 1 | 1 |
|  | K03799 | *htpX* | Heat shock protein HtpX | 1 | 1 | 1 |
| Drought and salt stress | K03313 | *nhaA* | Na+:H+ antiporter, NhaA family | 2 | 2 | 2 |
|  | K00931 | *proB* | Glutamate 5-kinase | 1 | 1 | 1 |
|  | K01881 | *proS* | Proline–tRNA ligase | 1 | 1 | 1 |
|  | K01546 | *kdpA* | Potassium-transporting ATPase subunit KdpA | 1 | 2 | 1 |
|  | K01547 | *kdpB* | Potassium-transporting ATPase subunit KdpB | 1 | 2 | 1 |
|  | K01545 | *kdpF* | Potassium-transporting ATPase subunit kdpF | 0 | 1 | 1 |
|  | K01548 | *kdpC* | Potassium-transporting ATPase subunit KdpC | 1 | 2 | 1 |
|  |  |  |  |  |  |  |
| *Genes associated with biofilm formation and motility*. | | | |  |  |  |
| **Metabolic Traits** | **Annotation Entry (KO)** | **Gene** | **Product Name** | **1SR** | **RZ5** | **RU18** |
| Biofilm formation | K11912 | *ppkA* | Serine/threonine-protein kinase PpkA | 1 | 1 | 1 |
|  | K11890 | *impM* | Type VI secretion system protein ImpM | 1 | 1 | 0 |
|  | K11891 | *impL* | Type VI secretion system protein ImpL | 2 | 2 | 0 |
|  | K11893 | impJ | Type VI secretion system protein ImpJ | 1 | 2 | 0 |
|  | K11902 | *impA* | Type VI secretion system protein ImpA | 1 | 1 | 0 |
|  | K11901 | *impB* | Type VI secretion system protein ImpB | 1 | 2 | 0 |
|  | K11900 | impC | Type VI secretion system protein ImpC | 1 | 2 | 0 |
|  | K11903 | *hcp* | Type VI secretion system secreted protein Hcp | 7 | 2 | 0 |
|  | K11895 | impH | Type VI secretion system protein ImpH | 1 | 2 | 0 |
|  | K11907 | vasG | Type VI secretion system protein VasG | 1 | 2 | 0 |
|  | K13488 | wspB | Chemotaxis-related protein WspB | 0 | 1 | 0 |
|  | K13489 | wspD | Chemotaxis-related protein WspD | 0 | 1 | 0 |
|  | K13487 | wspA | Methyl-accepting chemotaxis protein WspA | 0 | 1 | 0 |
|  | K13490 | wspE | Chemotaxis family, sensor histidine kinase and response regulator WspE | 0 | 1 | 0 |
|  | K13491 | wspF | Two-component system, response regulator WspF | 0 | 1 | 0 |
|  | K11444 | wspR | Two-component system, response regulator WspR | 0 | 1 | 0 |
|  | K21021 | tpbB | Diguanylate cyclase [EC:2.7.7.65] | 2 | 1 | 0 |
|  | K21022 | roeA | Diguanylate cyclase [EC:2.7.7.65] | 0 | 1 | 0 |
|  | K21023 | mucR | Diguanylate cyclase [EC:2.7.7.65] | 1 | 2 | 0 |
|  | K19291 | alg44 | Mannuronan synthase | 0 | 1 | 0 |
|  | K02356 | Efp | Elongation factor P | 2 | 1 | 1 |
| Flagellar protein | K03980 | murJ | Murein biosynthesis integral membrane protein murJ | 1 | 1 | 2 |
